# A large-scale functional analysis of genes expressed differentially in insulin secreting MIN6 sublines with high versus mildly reduced glucose-responsiveness

**DOI:** 10.1038/s41598-023-32589-2

**Published:** 2023-04-06

**Authors:** Aya Tanaka, Minami Kosuda, Midori Yamana, Asami Furukawa, Akiko Nagasawa, Midori Fujishiro, Genta Kohno, Hisamitsu Ishihara

**Affiliations:** Division of Diabetes and Metabolic Diseases, Nihon University School of Medicine, 30-1 Oyaguchikami-cho, Itabashi, 173-8610 Japan

**Keywords:** Cell biology, Endocrinology

## Abstract

Molecular mechanisms of glucose-stimulated insulin secretion (GSIS) from pancreatic β-cells are not fully understood. GSIS deteriorations are believed to underlie the pathogenesis of type 2 diabetes mellitus. By comparing transcript levels of 3 insulin secreting MIN6 cell sublines with strong glucose-responsiveness and 3 with mildly reduced responsiveness, we identified 630 differentially expressed genes. Using our recently developed system based on recombinase-mediated cassette exchange, we conducted large-scale generation of stable clones overexpressing such genes in the doxycycline-regulated manner. We found that overexpressions of 18, out of 83, genes altered GSIS. *Sox11* ((sex determining region Y)-box 11) was selected to confirm its roles in regulating insulin secretion, and the gene was subjected to shRNA-mediated suppression. While *Sox11* overexpression decreased GSIS, its suppression increased GSIS, confirming the role of *Sox11* as a negative regulator of insulin secretion. Furthermore, metabolic experiments using radiolabelled glucose showed *Sox11* to participate in regulating glucose metabolism. Our data suggested that overexpression screening is a feasible option for systemic functional testing to identify important genes in GSIS.

## Introduction

The prevalence of type 2 diabetes mellitus (T2DM) is rising worldwide. After the human genome project, over the past two decades, genetic analyses including genome wide association studies (GWAS) identified more than 400 genetic signals that might be associated with T2DM risk^1^. These genetic analyses have revealed heterogeneous origins of T2DM^2^, but the majority of variants were found to affect pancreatic β-cell function and/or β-cell fate determination. In parallel with the genetic analyses, molecular and cellular biological studies on insulin secreting cells identified numerous enzymes and transporters possibly regulating glucose-stimulated insulin secretion (GSIS)^3^. Thus, normal finely tuned GSIS is known to be generated on the basis of concerted actions of many gene products, probably more than 100. These lines of evidence are gradually clarifying the full picture of the mechanisms underlying insulin secretion, but a complete understanding is as yet lacking^3,4^. We still do not know precisely how the novel diabetes drug imeglimin augments insulin secretion, and several novel mechanisms have been postulated^5,6^. Ongoing identification of genes involved in regulating insulin secretion is one approach to fully elucidating insulin secretion mechanisms.

Genes that play important roles in deterioration of β-cell function in T2DM or that cause reduced β-cell glucose responsiveness have been identified in several ways: comparison of transcript levels between rodent insulin secreting cell lines^7–10^ or proteome analysis of rodent islets^11^ with low and high glucose-responsiveness, transcript or proteome profiling of human islets from diseased donors^12,13^ and more recently from metabolically defined living human pancreatic islet donors^14^. Candidate genes have also been obtained from studies of genomic risk variants identified through T2DM GWAS^15^. In these studies, several genes were tested for the functional impacts of altered gene expressions but these genes account for only a small portion of those playing roles in GSIS^12,16^. Genes are analyzed through suppression by lentivirus-mediated shRNA expression or the siRNA-mediated knockdown method. A systemic analysis of candidate genes located within 10 kbps around the risk mutations, found in GWASs, used the siRNA-mediated knockdown method^15^. However, custom preparation of siRNA for 100 or more genes would be very costly. More cost-effective methods for conducting large scale analyses are thus desired.

We recently reported establishment of a master insulin secreting MIN6-based cell line for efficiently generating stable transformants with a drug-inducible system^17^. Generating stable cell lines is somewhat laborious, but this cell line is very efficient and makes large-scale production of engineered clones possible, since it has a platform on the novel safe harbor genome locus for integration of foreign genes by means of recombinase-medicated cassette exchange (RMCE). Once generated, experiments can be carried out easily and repeatedly. Using this system, we conducted a large-scale analysis aimed at identifying genes important for insulin secretion.

We began by analyzing genes differentially expressed in MIN6 cell sublines with high versus mildly low glucose-responsiveness and thereby selected candidate genes. Then, we generated transformants overexpressing the candidate genes. Effects of gene expression on cellular phenotypes can be examined by loss-of-function and gain-of-function studies. Reducing or completely abolishing gene expression by gene silencing identifies genes that are necessary for a particular cellular function, while induced overexpression finds genes that are sufficient to generate a phenotype. As summarized by Prelich, overexpression studies have several advantages^18^. Most notably, this approach can identify regulatory rate-limiting steps and provides functional links even for redundant genes. In addition, as an experimental technique, overexpression is economical. However, it must be recognized that overexpressions can lead to identification of artificial effects that are not physiologically relevant, including those due to excess protein expressions, as already reported in detail^19,20^. Keeping these factors in mind, we have endeavored to test the effects of overexpressing more than eighty candidate genes.

## Results

### Transcriptome analyses in MIN6 cell sublines with high and mildly low glucose responsiveness

**Figure 1:**
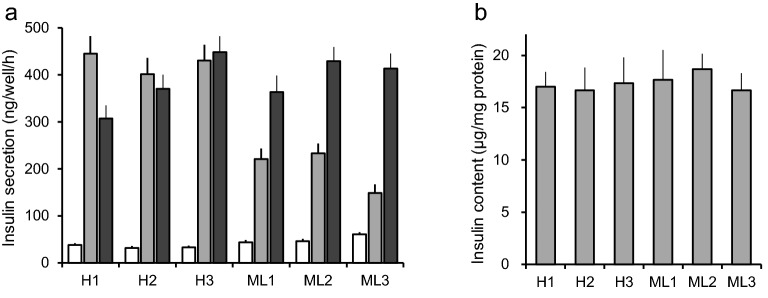
MIN6cl4 sublines with high and mildly low glucose-responsiveness. (**a**) Insulin secretion evoked by glucose (5 mM, white bars; 20 mM grey bars) or 30 mM KCl (black bars) from sublines with high glucose-responsiveness (H1, H2, H3) and from sublines with mildly low glucose-responsiveness (ML1, ML2, ML3). Data are presented as means ± SE, n = 3 wells. The results shown are representative of two independent experiments with similar results. (**b**) Insulin contents in MIN6cl4 sublines. Data are presented as means ± SE, n = 3 wells. The results shown are representative of two independent experiments with similar results.

While generating MIN6 cell sublines expressing the tetracycline-inducible transcription factor Tet3G (Takara-Clontech, Shiga, Japan)^17^, we found GSIS to differ among sublines. The expression levels of Tet3G itself are regarded as not affecting GSIS. We evaluated these sublines based on the glucose-responsiveness of insulin secretion at 20 mM versus that at 5 mM. We selected three sublines which showed a ratio higher than tenfold (high responder, the H group), and three sublines with ratios between 2 and fivefold (mildly low responder, the ML group) (Fig. 1a). Insulin contents did not differ among these 6 sublines (Fig. 1b). The subline H3 is the MIN6Tet3G9 cell line, the parental subline of MIN6CE40 and MIN6CEon1^17^.

We compared transcript levels of these two groups by microarray using the Agilent Mouse SurePrint G3 Array (Supplementary File 1). We first selected genes based on their average expression levels differing by twofold between groups H and ML. Considering that directional consistency in the group is important, we then eliminated genes if their expression directions differed among the three sublines within each group. Finally, we only selected annotated genes. This selection process identified 262 genes highly expressed in the H group and 368 genes highly expressed in the ML group (Supplementary Tables S1 and S2). These included 6 genes which had been identified through comparison between MIN6cl4 cells and long-passaged MIN6 cells, as reported previously^17^. We checked these genes individually in PubMed focusing on their involvement in GSIS. We initially found that 25 genes out of 630 had already been reported as influencing insulin secretion. During the course of this project spanning almost 8 years, another 38 genes have been reported as GSIS regulators (Supplementary Tables S3 and S4). Among the total 63 genes, there were 30 genes with higher expressions in the H group and 33 with higher expressions in the ML group. These data suggested that our approach was promising for identifying novel genes important for insulin secretion. In addition, 16 out of 631 selected genes have already been reported to contribute to the β-cell stress response and/or mass regulation (Supplementary Table S5).

For these 630 genes, we conducted a gene ontology analysis but found no differences between the two groups. Because one of our purposes was to ascertain how efficiently direct cellular engineering analyzes candidate genes, we did not additionally analyze the microarray data, instead proceeding to cellular experiments. From the 630 genes, we chose 100 genes for overexpression screening, in order to test whether or not such a screening strategy is efficient. Since assessing the discovery rate of novel GSIS regulators was not our purpose, we did not select genes based on a solid principle, instead making the choice based on their molecular functions (regulating intracellular metabolism or ion channels, and so on) or because differences in their expression levels were more than threefold between the H and ML groups. In addition, *Cited 4*, *Arhgef3*, and *Folr1*, which were tested in our previous study^17^, were also chosen, to confirm the reproducibility of overexpression experiments.

### Large-scale functional analysis of candidate genes possibly regulating insulin secretion

**Figure 2:**
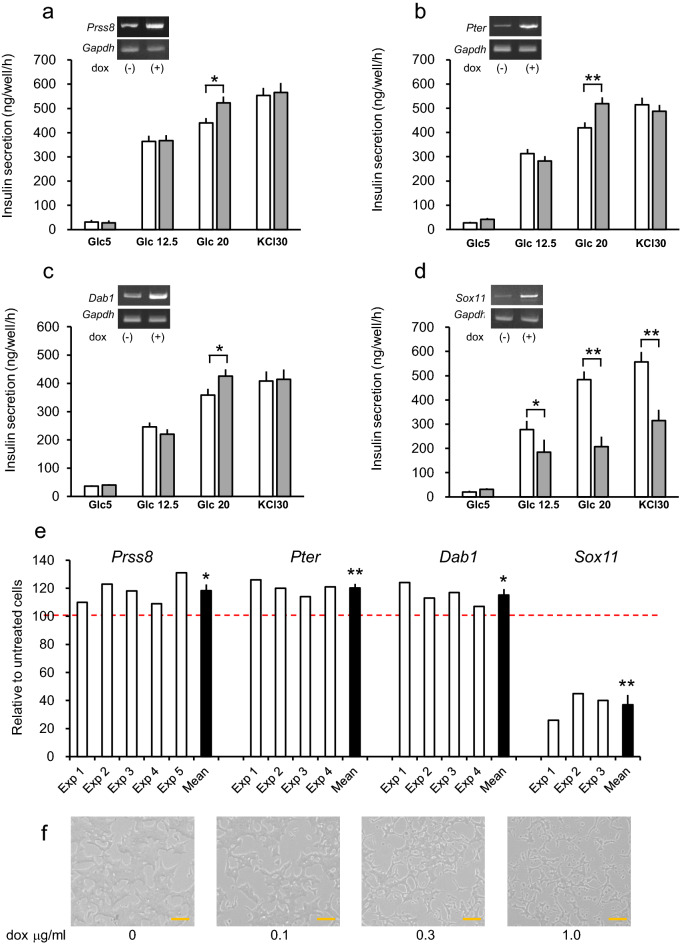
Examples of overexpression screening. (**a**–**d**) Effects on insulin secretion of the overexpressions of 4 genes (**a** *Prss8*; **b** *Pter*; **c** *Dab1*; **d** *Sox11*) differentially expressed in highly and poorly glucose-responsive MIN6 cells. Overexpressions were induced by treatment with dox for 2 days as shown in the inset for RT-PCR gel results. Insulin secretions from dox-treated and untreated cells are shown with grey and white bars, respectively. Graphs are representative individual experiments and data are means ± SE, n = 3 wells. *P < 0.05, **P < 0.01, Student unpaired *t* test. Uncropped RT-PCR data are provided in Supplementary Fig. S2. (**e**) Summary of the insulin secretion results for 4 genes at 20 mM glucose. Amounts of secreted insulin are expressed as values relative to those from dox-untreated cells at 20 mM glucose, which is defined as 100 (dotted red line). Insulin secretion data at 20 mM glucose relative to those from untreated cells are presented for individual experiments (white bars) and for their averages (black bars, means ± SEM). Mean values are tested for significance from untreated cells using a one-sample *t* test. *P < 0.05, **P < 0.01. (**f**) Effects on cell number of the *Sox11* overexpression. Overexpression was induced by treatment with different concentrations of dox for 2 days. Bars 100 μm.

To generate efficiently stably-transfected MIN6 cells with the drug-induced system, we recently generated MIN6CEon1 cells^17^. In this cell line, the platform accepting foreign genes by means of RMCE method has intentionally been placed at the genome locus 10.5 kbps upstream from the *Zxdb* initiation codon. This locus was identified in the MIN6CE clone 40 (MIN6CE40), the prototype cells of MIN6CEon1 cells, in which a similar platform was placed through random integration^17^. We used both cells for the present study. Complimentary cDNAs were cloned from MIN6 cell total RNA and subcloned into either pF3BsdTREFwr or pF3HygTREFwr donor plasmids^17^, and we then transfected these MIN6 master cells having cassette exchange platforms. After appropriate selection with antibiotics, the cells were used for GSIS assay. Since nearly 90% of MIN6CE40 and essentially 100% of MIN6CEon1 cells underwent cassette exchange, pooled cells were considered to be sufficient for screening. After confirming doxycycline-induced expression of cDNAs of the genes of interest (GOI) by RT-PCR (Fig. 2a–d), GSIS assays were conducted.

In total, nearly 100 stable clones were generated. During the course of this study, 9 genes out of the 100 selected genes were reported to be GSIS regulators (Supplementary Tables S3 and S4). In addition, we have failed to clone cDNAs of 8 genes. Therefore, the results of overexpressions of 83 genes are presented. Each gene was overexpressed by addition of 1 μg/ml doxycycline (dox) for 2 days and GSIS analysis was performed 3 to 5 times for each gene. We have tested 5 mM glucose, 12.5 mM glucose, 20 mM glucose, and 5 mM glucose + 30 mM KCl for the tests. KCl has an advantage, for testing, in that it provides a hint as to whether or not insulin secretion signaling after Ca^2+^ entry is altered. When it changes, final exocytosis mechanisms are likely altered or the insulin content itself may even be altered. Therefore, when the KCl response was observed to change, the insulin content was analyzed. Examples of insulin secretion results of individual representative experiments are presented for *Prss8* (protease, serine 8, Fig. 2a), *Pter* (phosphotriesterase-related, Fig. 2b), *Dab1* (disabled 1, Fig. 2c) and *Sox11* ((sex determining region Y)-box11), Fig. 2d).

Insulin secretion at the basal glucose concentration (5 mM) was very low and no differences were observed between dox-untreated and -treated MIN6 cells harboring 4 different cDNAs. Since differences were apparently observable at 20 mM glucose, but not at 5 mM and 12.5 mM glucose concentrations, we have focused on insulin secretion at 20 mM glucose. (For the same reason, screening experiments were conducted at 5 mM, 20 mM glucose and 30 mM KCl at later stages). Figure 2e shows the results of insulin secretion at 20 mM glucose relative to those from dox-untreated control cells in individual tests for these genes. Overexpressions of *Prss8, Pter* and *Dab1* increased insulin secretion, while that of *Sox11* reduced insulin secretion, in response to 20 mM glucose. KCl-stimulated insulin secretion was unaltered in cells overexpressing *Prss8, Pter* and *Dab1*, but was reduced in *Sox11*-overexpressing cells. In fact, *Sox11* overexpression lowered MIN6 cell numbers dose-dependently, as shown in Fig. 2f. Insulin contents in dox (1 μg/ml)-treated *Sox11* overexpressing cells were 56 ± 7% of those of dox-untreated control cells (*p* < 0.01, n = 4 experiments).

**Figure 3:**
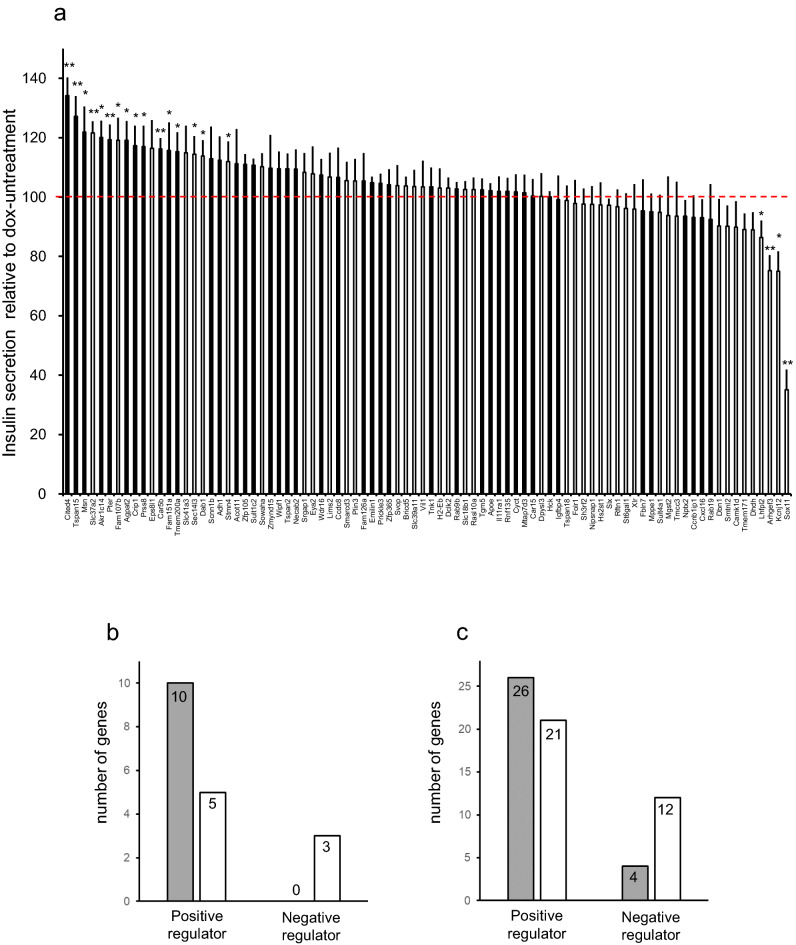
Summary of large-scale analysis of overexpression effects of extracted genes. (**a**) Effects of overexpressions on insulin secretion at 20 mM glucose are summarized. Insulin secretion from dox-treated overexpressing cells relative to that from dox-untreated cells at 20 mM glucose (defined as 100, dashed line) are presented. Insulin secretion analyses were performed 3 or 5 times for each gene. Black and white columns represent genes which had been found in the previous microarray analysis to be preferentially expressed in the H and ML groups, respectively. Data are means ± SEM. Mean values were tested for significance using a one-sample *t* test. *P < 0.05, **P < 0.01. (**b**,**c**) Grey bars represent numbers of genes preferentially expressed in MIN6 cells with high glucose-responsiveness and white bars those with mildly low glucose-responsiveness, as identified by the functional screening (**b**) and by literature search (**c**).

Overall results are summarized in Fig. 3a. In addition to the *Cited 4* and the *Arhgef3* genes reported previously^17^, overexpressions of 15 genes increased while those of 3 genes decreased insulin secretion in response to 20 mM glucose. Thus, our overexpression screening identified 18 genes as GSIS regulators, 15 positive and 3 negative regulators. Of the 15 positive regulators, 10 genes were highly expressed in MIN6 sublines with high glucose-responsiveness and 5 in mildly low responsiveness. All 3 with a suppressive effect (*Lhfpl2, Kcnj12, Sox11*) were overexpressed in the ML group (Fig. 3b). Interestingly, our literature search on 630 differentially expressed genes revealed similar tendencies. The search found 47 positive regulators and 16 negative regulators. Positive regulators were present in nearly equal amounts in the H and ML groups (26 genes in the H group and 21 in the ML group), while negative regulators belonged mainly to a gene group overexpressed in low-responsive sublines (4 genes in the H group and 12 in the ML group) (Fig. 3c).

### Validation of *Sox11* as a regulator of GSIS

**Figure 4:**
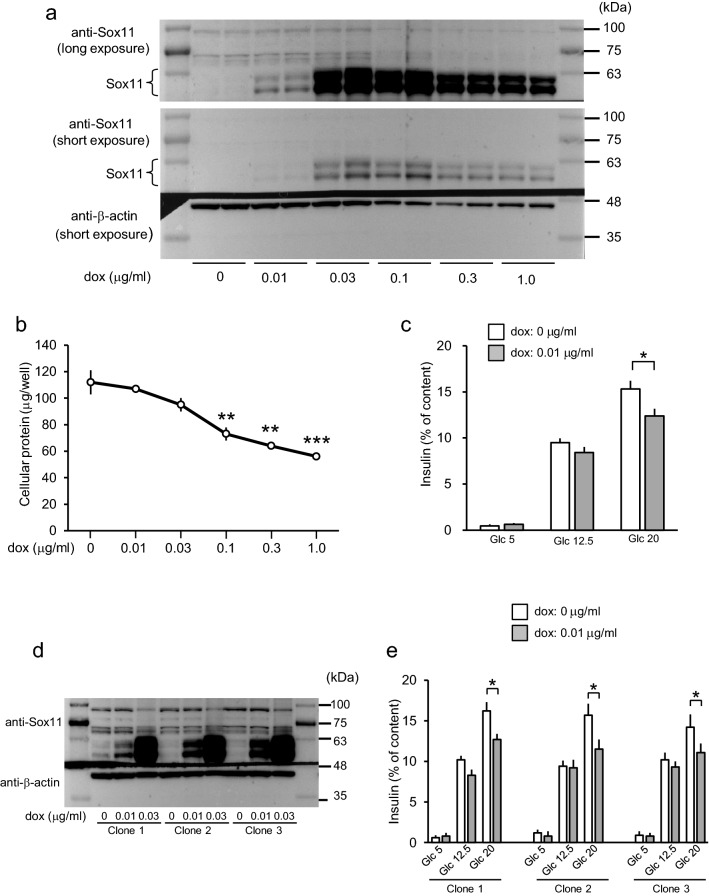
Doxycycline dose-dependent effects on *Sox11* overexpression. (**a**) MIN6 cells harboring the tetracycline-dependent *Sox11* expression unit were cultured for 2 days with different concentrations of dox and subjected to Western blot analysis. Membranes were cut just above the 48 kDa marker levels and probed with anti-Sox11 or anti-β-actin antibody. The representative blot shown is from 3 experiments. Uncropped images are presented in Supplementary Fig. S3. (**b**) MIN6 cells treated as in (**a**) were dissolved with 1N NaOH and protein concentrations were determined with the Pierce protein assay reagent. Data are means ± SEM, n = 3 experiments. **P < 0.01, ***P < 0.001. (**c**) GSIS from 0.01 μg/ml dox-treated and -untreated MIN6 cells harboring the *Sox11* expression unit. Data are presented as means ± SEM, n = 3 experiments. *P < 0.05. (**d**) Western blot analyses were performed using subclones overexpressing Sox11 as described in (**a**). The representative blot shown is from 2 experiments. Uncropped images are presented in Supplementary Fig. S5. (**e**) GSIS from 0.01 μg/ml dox-treated and -untreated subclones harboring the *Sox11* expression unit. Data are presented as means ± SEM, n = 3 experiments. *P < 0.05.

Among these genes, we particularly focused on *Sox11*, because in addition to overexpression of this gene significantly reducing GSIS, *Sox11* expression is reportedly enriched in human fetal β-cells with glucose-unresponsiveness^21^, and because compromised cell viability upon *Sox11* overexpression was also reported in some retinal ganglion cells^22^. We first evaluated the dose-responses relationship of the dox effect on *Sox11* overexpression (Fig. 4a), because 1 μg/ml dox caused substantial MIN6 cell death, making it difficult to precisely evaluate insulin secretion response. Treatments with dox at doses as high as 0.03 μg/ml did not reduce cellular protein contents (Fig. 4b) and thus cell viability. At 0.01 μg/ml dox, *Sox11* expressions were increased and 20 mM glucose induced insulin secretion was suppressed in dox-treated cells (Fig. 4c): in order to show glucose-responsiveness in insulin secretion clearly and to achieve normalization among experiments, insulin secretion results are shown as relative amounts of secreted insulin to cellular content.

Data presented above were obtained using pooled cell populations. Such a strategy, instead of using subclones, was employed in previous studies^23,24^. When using stable clones, clonal variabilities are problematic, but pooled polyclonal cell populations were considered to represent the average of clones, and shown to reliably evaluate the effects of genetic manipulations^23^. Since clones with a growth advantage might well be the major populations after long culture periods, they should be used within limited periods. In this study, all pooled cells were used within a month after thawing fresh aliquots of cells. In addition, we minimized clonal variations by using isogenic cells generated from our own master cell lines, either MIN6CE40 or MIN6CEon1, by the RMCE method^17^. Subclones generated by the RMCE method based on a master cell line reportedly showed low clonal variabilities during three-month cultivation^25^. Furthermore, we used a tetracycline-inducible system. Using the inducible system, the presence or absence of dox for less than 1 week is the sole difference between control and analyzed cells. Thus, observed phenotypic differences are thought to be attributable mainly to induction of changes in expression of the sequence of interest and thus to be minimally affected by cellular heterogeneity. To confirm results obtained using pooled cell populations, we studied GSIS in three independent inducible *Sox11* clones. These clones exhibited similar inducible expression of Sox11 protein (Fig. 4d). In addition, glucose (20 mM)-stimulated insulin secretion was suppressed in all three clones with an increased expression of Sox11 in response to 0.01 μg/ml dox treatment (Fig. 4e), an observation similar to those made in a pooled cell population (Fig. 4c).

**Figure 5:**
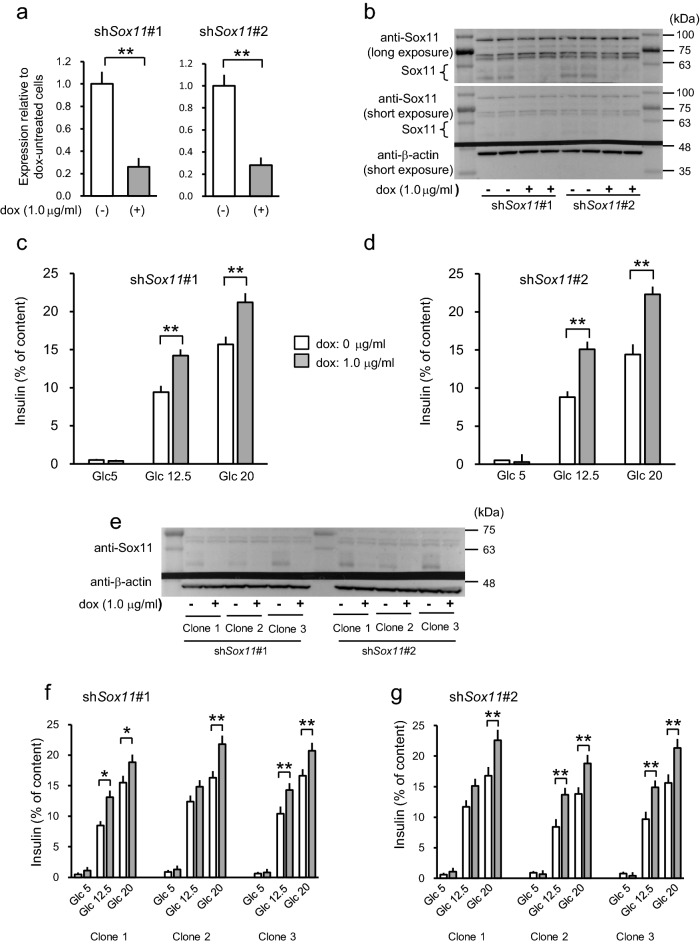
Effects of suppression of *Sox11* expressions on insulin secretion. (**a**) RT-PCR analyses of the effects of expressing two different shRNAs for targeting *Sox11,* sh*Sox11*#1 and sh*Sox11*#2 . Data are means ± SEM, n = 3 experiments. **P < 0.01. (**b**) MIN6 cells harboring the tetracycline-dependent shRNA against *Sox11* expression unit (sh*Sox11*#1 and sh*Sox11*#2) were cultured with 1 μg/ml of dox for 6 days and subjected to Western blot analysis. Membranes were analyzed as described in the legend for Fig. 4a. Shown is the representative blot from 3 experiments. Uncropped images are presented in Supplementary Fig. S4. (**c**,**d**) Glucose-stimulated insulin secretion from cells with reduced expressions of *Sox11,* sh*Sox11*#1 (**c**) and sh*Sox11*#2 (**d**). Data are means ± SEM, n = 3 experiments.  **P < 0.01. White bars represent dox-untreated control cells and grey bars dox (1 μg/ml) -treated shRNA expressing cells. (**e**) Western blot analyses were performed using subclones expressing sh*Sox11*#1 and sh*Sox11*#2 as described in (**b**). Shown is the representative blot from 2 experiments. Uncropped images are presented in Supplementary Fig. S6. (**f**, **g**) Glucose-stimulated insulin secretion from subclones with reduced expressions of *Sox11,* sh*Sox11*#1 (**f**) and sh*Sox11*#2 (**g**). Data are means ± SEM, n = 3 experiments. *P < 0.05, **P < 0.01. White bars represent dox-untreated control cells and grey bars dox (1 μg/ml) -treated shRNA expressing cells.

Then, we attempted to suppress expressions of the *Sox11* gene by expressing shRNA. We selected two target sites through the shERWOOD web site. We initially employed pooled cell populations expressing either one of the sh*Sox11* sequences. Although *Sox11* expression was very low in MIN6CEon1 cells, shRNA expression with 1 μg/ml dox for 6 days caused further decreases in its transcript levels (Fig. 5a) as well as protein levels (Fig. 5b). Suppression of *Sox11* increased insulin content (21.7 ± 1.2 μg/mg protein vs. 23.9 ± 1.0 for dox-untreated and -treated cells, p < 0.05, n = 3 experiments). As shown in Fig. 5c,d, shRNA-mediated suppression of *Sox11* increased insulin secretion, verifying its roles as a negative regulator of GSIS. Although *Sox11* suppression increased insulin contents, cell numbers did not change (data not shown). Subsequently, we examined 3 independent clones expressing either one of the sh*Sox11*#1 and #2 sequences. Similar effects on Sox11 protein levels (Fig. 5e) and effects on GSIS (Fig. 5f,g) were observed in these three independent clones expressing shRNA sequences.

**Figure 6:**
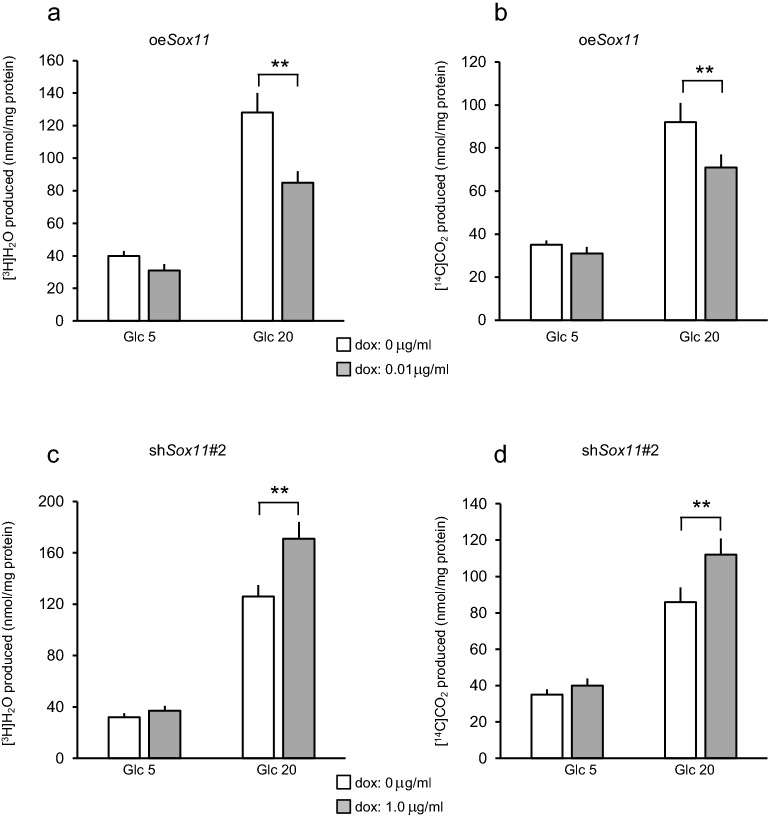
Effects of overexpression or suppression of *Sox11* on glucose metabolism. (**a**,**b**) Glucose utilization estimated using [5-^3^H]glucose (**a**) and glucose oxidation estimated using [U-^14^C]glucose (**b**) in cells with *Sox11* overexpression (oe*Sox11*). White bars represent dox-untreated control cells and grey bars dox (0.01 μg/ml)-treated shRNA expressing cells. Data are means ± SEM, n = 3 experiments.   **P < 0.01. (**c**,**d**) Glucose utilization (**c**) and glucose oxidation (**d**) in cells with *Sox11* suppression. Results of *Sox11* suppression are presented in cells expressing sh*Sox11*#2. White bars represent dox-untreated control cells and grey bars dox (1 μg/ml)-treated shRNA expressing cells. Data are means ± SEM, n = 3 experiments.    **P < 0.01.

An advantage of generating stable clones is that one can easily proceed to further experiments requiring that the majority of cells be engineered, such as metabolic studies. Thus, we conducted metabolic studies using radiolabeled glucose in polyclonal cell populations. As shown in Fig. 6a, glycolytic flux estimated by generation of [^3^H]H_2_O from [5-^3^H]glucose was decreased by *Sox11* overexpression. In addition, glucose oxidation mainly in mitochondria estimated by generation of [^14^C]CO_2_ from [U-^14^C]glucose was diminished by *Sox11* overexpression (Fig. 6b). Conversely, suppression of *Sox11* expression by sh*Sox11*#2 expression increased production of [^3^H]H_2_O (Fig. 6c) and [^14^C]CO_2_ (Fig. 6d). Similar results were obtained in MIN6CEon1 cells expressing sh*Sox11*#1 (data not shown). These results indicated *Sox11* to modulate glucose metabolism, thereby regulating insulin secretion.

## Discussion

In this study, we have endeavored to conduct a large-scale investigation of candidate genes possibly modulating insulin secretion, which had been identified by comparing two sets of MIN6 insulinoma cell sublines, 3 highly responsive and 3 with mildly lowered glucose-responsiveness. To date, systemic analyses of candidate genes regulating insulin secretion have been conducted in only one study, employing synthetic RNA for siRNA-mediated suppression of gene expression^15^. Our study provides a feasible option for systemic functional testing of genes, modulating the expression of which might alter insulin secretion.

In order to select candidate genes which might play an important role in insulin secretion, especially in response to elevated glucose concentrations, we compared transcript levels between sublines with high glucose-responsiveness and sublines with moderately low responsiveness. We found 630 annotated protein coding genes to be differentially expressed. Of these, 25 genes at the beginning of this project almost 8 years ago and 63 at the stage of manuscript preparation had been reported to play important roles in insulin secretion (Supplementary Tables S3 and S4). Other studies also examined genes which were differentially expressed in high responder and low responder MIN6 cells^7–10^. We compared genes extracted in this study to those found in studies by other investigators (Supplementary Fig. S1). Minami et al*.*^7^, the earliest study, reported only 9 genes, but none of them are common with our 630 genes. O’Driscoll et al. reported categories of genes and only several representative genes but not entire individual genes that were differentially expressed. Therefore, we compared our 630 genes with those reported by Lilla et al.^8^ (201 genes) and Yamato et al.^10^ (75 genes). As shown in the Supplementary Fig. S1, we found only 19 genes and 5 genes that were common to our investigations and those of Lilla et al. and Yamato et al*.*, respectively. Eleven genes are common between studies by Lilla et al. and Yamato et al. Interestingly, *Sox11* is the single gene common to all three studies. The reason for these low rates of genes in common between studies is not clear at present. Different methods and/or microarrays used to extract differentially expressed genes might cause variations. Another reason might involve differences in cells used, especially, low responder cells. In the previous studies, very poorly responsive cells were used as low responder cells, as summarized in the Supplementary Table S6. In the present study, we assumed that loss of glucose responsiveness is one of the early changes preceding entire loss of differentiated phenotypes of insulin secreting cells. In the advanced stage of dedifferentiation, expression levels of many genes would be changed. These changes would mask changes in genes related to GSIS. This is why we selected sublines with moderately low glucose-responsiveness.

Which is the more efficient approach, loss-of-function or gain-of-function studies, for finding genes important in GSIS? Both study types have merits and demerits. Loss-of-function studies can identify genes that are necessary for a particular cellular function, while gain-of-function studies points to genes that are sufficient to generate a phenotype. For example, GLUT2 does not play a regulatory role in GSIS, while GSIS is regulated by glucokinase, catalyzing the glucose phosphorylation step. Therefore, glucose metabolism and glucose-stimulated insulin secretion are increased by overexpression of glucokinase^26,27^, but not GLUT2^28^. Nonetheless, homozygous knockout of GLUT2 caused impaired insulin secretion^29^. Therefore, overexpression studies are useful for identifying regulatory steps, while loss of function studies are appropriate for identifying genes which are involved in an important pathway mediating a phenomenon, GSIS in the present case. In addition, overexpression experiments are easy to perform. Producing expression vectors with cDNA cloned employing the RT-PCR method with a pair of primers is cheaper than purchasing effective siRNAs. Nevertheless, overexpression studies may yield artificial effects, which may lead to misunderstanding of the results.

Employing large-scale stable clone generation, we identified 18 out of the 83 genes tested as possible modulators of GSIS. Although this rate is lower than that of the siRNA-based loss-of-function screening previously reported^15^, identification of more than 20% of the genes tested is efficient. Of the 15 genes positively regulating GSIS, 10 genes were among genes with expression levels higher in the H group than in the ML group. These genes are considered to be directly involved in the highly glucose-responsive phenotype. In addition to the *Arhgef3* previously reported^17^, *Kcnj12*, *Sox11*, and *Lhfpl2* overexpressions decreased GSIS in MIN6CE cells, and thus are considered to be negative regulators of GSIS. Since expressions of these genes were increased in the ML sublines, these genes may also be directly involved in the lower glucose-responsive phenotype of the ML sublines. In contrast, very interestingly, overexpressions of 5 (the *Dab1*, *Fam107b, Sec14l3, Stmn4* and *Slc37a2*) genes increased GSIS. Although overexpression tests suggest that these genes are positive regulators of GSIS, they are up-regulated in low glucose-responsive sublines. As shown in the Supplementary Table S4, by searching the literature, we recognized that expressions of 21 genes that are known to be positive regulators of GSIS, including *Adcy5*, *Robo3* and *Wnt4*, were up-regulated in the ML sublines. Although the precise mechanisms underlying the up-regulations of these positive regulators in the ML sublines have yet to be identified, if the ML sublines are in the early phase of losing differentiated phenotypes of insulin secreting cells, we can reasonably speculate that up-regulating these genes would be a counter-response that might maintain glucose-responsiveness.

Among the aforementioned 18 genes, we were especially interested in *Sox11* and generated stable cell lines expressing shRNA against this gene. Note that *Sox11* is the only gene common to previous MIN6 transcript studies and the present study (Supplementary Fig. S1). Effects of shRNA-mediated silencing of *Sox11* gene increased glucose-responsiveness, confirming the roles of *Sox11* as a negative regulator in GSIS. In addition, *Sox11* overexpression caused cell death. Similar cell-killing effects of *Sox11* overexpression have been reported in some retinal ganglion cells^22^. We also found that *Sox11* modulates glucose metabolism, consistent with the results of decreased metabolic activity found in a pro B-cell line overexpressing *Sox11*^30^. The primary regulator of glycolysis in β-cells is glucokinase. However, regulation of glycolysis in the β-cell is more complex, as recently demonstrated by the observation that supplying Pck2-mediated phosphoenolpyruvate enhances glucose metabolism^31^. In addition, regulation of glucokinase activities is multifold^32^. Further analyses of these cell lines will clarify mechanisms by which *Sox11* modulates glucose metabolism. *Sox11* is expressed in the fetal β-cells but only at very low levels in adult β-cells^21,33^ and its misexpression has also been reported in immature stem-cell derived β-cells with low glucose-responsiveness^21,34^. Interestingly, defects in glycolysis are reportedly associated with their low insulin secretory responses^35^. It is possible that *Sox11* misexpression plays a role in the immature phenotype. In addition, *Sox 11* may play an important role in development of the pancreas, because *Sox11*-deficient embryos reportedly exhibit pancreatic hypoplasia^36^. Precisely when *Sox11* expression is switched on and off of during the fetal and adult periods might be important for both GSIS and cell survival.

The present study has several limitations. First, since we did not choose genes among the 630 extracted through transcript levels by applying a valid principle, whether the rate of identifying novel regulators of GSIS can be applied to the remaining approximately 500 genes and/or to studies with similar approaches is not clear. Second, we did not confirm overexpressions at protein levels, such that it is possible that important genes exist but were not identified as GSIS regulators in this study because of insufficient overexpression at the protein levels. Lack of validation of protein levels is a common limitation in large scale analysis^15^ and this issue needs to be resolved in the near future. Third, we focused on GSIS at 20 mM, since overexpression did not alter GSIS at an intermediate, i.e., a more physiologically relevant, glucose concentration. Thus, physiological roles of these genes must be carefully interpreted.

In summary, using a newly established MIN6 master cell line, we performed a large-scale functional analysis of candidate modulators of GSIS in this study and identified 18 genes possibly playing important roles in GSIS. As overexpression experiments can produce artificial phenomena, we must be careful and will need to conduct complimentary knockdown experiments for confirmation. Although we have eliminated functional non-coding RNA, the known roles of which in cellular processes are expanding, we recognize the importance and plan to test these components of genetic regulators in the near future. The discovery of new players in GSIS may allow the initiation of studies aimed at revealing how such molecules function in concert with known signaling pathways. It is hoped that elucidating these players and connections will make it possible to obtain a full understanding of the complex processes governing GSIS.

## Methods

### Cell culture

MIN6CEon1 cells or MIN6CE40 cells, a prototype cell line of MIN6CEon1, were previously described^17^ and cultured in DMEM, the same method as that used for parental MIN6 cells^37,38^.

### Microarray analysis

Total RNA was extracted from MIN6 cell sublines using the RNeasy kit (Qiagen) and was amplified and labeled with Cyanine 3 (Cy3) using an Agilent Low Input Quick Amp Labeling Kit, one-color (Agilent Technologies, Palo Alto, CA) following the manufacturer's instructions. For each hybridization, 1.65 μg of Cy3 labeled cRNA were hybridized at 65 °C for 17 h to an Agilent Mouse GE 8x60K Microarray (Design ID: 028005). After washing, the microarrays were scanned using an Agilent DNA microarray scanner. In total, there were of 55,681 probes on the Agilent Mouse GE 8x60K Microarray (Design ID: 028005) without control probes.

### RT-PCR cloning of cDNAs

Total RNA extracted from MIN6 cells were subjected to cDNA synthesis employing ReverTra Ace (Toyobo Life Science, Tokyo, Japan) with a mixture of random and oligodT primers. PCR was performed using Q5 DNA polymerase (New England Bio Labs (NEB), Ipswich, MA). cDNA fragments were cloned into pF3BsdTreGfpFwr or pF3HygTreGfpFwr^17^ using T4DNA ligase (Quick ligation kit from NEB) after digestion with SalI and EcoRI. We also employed the InFusion cloning method (Takara) for cDNA cloning. Primers custom-ordered to Integrated DNA Technologies (Coralville, Iowa) are shown in the Supplementary Table S7.

### Construction of plasmid for knockdown by microRNA-embedded shRNA

Plasmids for knockdown were constructed as described previously^17^. Target sequences were selected by using the shERWOOD website (http://sherwood.cshl.edu:8080/sherwood/) and were as follows; sh*Sox11*#1, 5′-GACGACCTCATGTTCGACCTGA-3′; sh*Sox11*#2, 5′-CGGCTCTACTACAGCTTCAAGA-3′.

### Electroporation and generation of clonal cell lines and polyclonal cell populations

3 × 10^6^ MIN6CEon1 cells or MIN6CE40 cells were electroporated with pCAG-Flpe^39^ and pF3BsdTreGOIFwr or pF3HygTreGOIFwr using the Nucleofector^®^ (Lonza, Allendale, NJ, USA) with the preset program G-16. Cells were suspended in 7 ml of culture medium and divided into 1, 2, and 4 ml portions in 10-cm dishes and medium was then added up to 8 ml. Four days later, blasticidin (2.5 μg/ml) or hygromycin (200 μg/ml) added to the cells, which were then allowed to grew for 3 weeks. Individual colonies were picked up from a dish onto which 1 ml of electroporated cells had been seeded and pooled polyclonal cells were generated by gathering colonies in a dish seeded with 4 ml electroporated cells. Then, the cells were expanded, frozen and stored in liquid nitrogen. All of the experiments were performed within a month after thawing a fresh aliquot of cells.

### Western blot

Cells were dissolved in SDS sample buffer and proteins were subjected to SDS-PAGE and then transferred to nitrocellulose membranes. Membranes were probed with rabbit anti-Sox11 antibody (1:500) (HPA000536; Atlas Antibodies) or with mouse anti-β-actin antibody (1:5000) (#60008-1-Ig, Proteintech) overnight at 4 °C, and then incubated for 1 h with donkey anti-rabbit IgG (1:10,000) or with sheep anti-mouse IgG (1:10,000) conjugated with horseradish peroxidase (GE Healthcare, Piscataway, NL, USA). Detection was accomplished employing EZWestLumi plus reagent and visualized using WSE-6200HLuminoGraphII (ATTO, Tokyo, Japan).

### Static insulin secretion assays

For insulin secretion, cells (1 × 10^5^/well) were seeded in 24-well plates and subjected to dox treatment (1 μg/ml). The cells were pre-incubated in HBKRBB (119 mM NaCl, 4.74 mM KCl, 2.54 mM CaCl_2_, 1.19 mM MgC1_2_, 1.19 mM KH_2_PO_4_, 25 mM NaHCO_3_, 10 mM Hepes, pH 7.4; HBKRBB) with 0.1% bovine serum albumin and 5 mM glucose for 0.5 h, and then incubated with HBKRBB with either 5, 12.5 mM glucose, 20 mM glucose, or 5 mM glucose + 30 mM KCl. After a one-hour incubation period, the media were collected and assayed for immunoreactive insulin by ELISA (Mercodia, Uppsala, Sweden, #10-1247-10 or #10-1250-10). Secreted insulin levels were compared directly or after normalization with the cellular insulin content. Insulin content was assayed after extraction with acid ethanol.

### Measurement of glucose metabolism using [5-^3^H]glucose and [U-^14^C]glucose

Glucose utilization was measured by following the conversion of [5-^3^H]glucose into [^3^H]H_2_O as previously described^38^. Cells were seeded in 24-well plates and subjected to dox treatment. The cells were pre-incubated in HBKRBB with 0.1% bovine serum albumin and 5 mM glucose for 0.5 h, and then incubated with HBKRBB with 5, 12.5, or 20 mM [5-^3^H]glucose. After a 2-h incubation period, a 0.1 ml aliquot of the incubation medium was transferred to a microtube and then placed in plastic scintillation vials containing 0.6 ml of distilled water. The vials were stoppered and kept at 37 °C for 36 h to allow the [^3^H]H_2_O in the microtube to equilibrate with the water. Subsequently, the microtube was taken out and 10 ml of scintillation fluid were added.

Glucose oxidation, which occurred mainly in the mitochondria, was measured by following the conversion of [U-^14^C]glucose into [^14^C]CO_2_ following published methods^40,41^. Cells were seeded in 24-well plates and subjected to dox treatment. The cells were pre-incubated in HBKRBB containing 5 mM glucose for 0.5 h, then incubated with HBKRBB with 5, or 20 mM [U-^14^C]glucose. A rubber plate with the same scale, which has 24 holes each 10-mm in diameter, was placed on the well plate. On a hole of the rubber plate, we placed a piece of filter paper (13 mm × 13-mm) containing 1 M KCl. Then, the lid of the 24-well plate was set and clamped, and kept at 37 °C for 2 h. Subsequently, the filter paper was transferred to plastic scintillation vials and 10 ml of scintillation fluid were added.

### Literature search

We checked for the selected genes by conducting a search in PubMed search. “Gene name” and “islet”, “Gene name” and “beta-cell”, “Gene name” and “insulin”, “Gene name” and “diabetes” were the terms used.

### Statistical analysis

Data in Fig. 1a,b are presented as means ± SE. For Fig. 2a–d, data are presented as means ± SE. For Fig. 2e, white bars (results of individual experiments) represent means of wells, and black bars (average of individual experiments) represent means ± SEM. Normality was tested using the Shapiro–Wilk test. Figures 2e and 3 show the results of analyzing insulin secretion at 20 mM glucose in dox-treated cells overexpressing test genes relative to those in untreated cells are analyzed using a one-sample *t* test. Data are presented as means ± SEM for Figs. 4 and 5. Statistical significance was tested using the unpaired Student’s *t* test. All analyses were performed with IBM SPSS Statistics for Windows Version 25 J (IBM corp., Armonk, NY, USA).

## Supplementary Information

Supplementary Information 1.

Supplementary Information 2.

## Data Availability

All data provided in the article and the Supplementary file are available from the corresponding author on reasonable request.
