# Supplementary material for: A large-scale functional analysis of genes expressed differentially in insulin secreting MIN6 sublines with high versus mildly reduced glucose-responsiveness
Source: Sci Rep. 2023 Apr 6;13:5654. doi: 10.1038/s41598-023-32589-2 (PMC10079668; doi:10.1038/s41598-023-32589-2)

List of genes preferentially expressed in sublines with high glucose responsiveness selected by our criteria described in the text

	Annotation	Normalized Data					
	GeneSymbol	H1	H2	H3	ML1	NL2	ML3
1	Msn	1.45	1.58	3.28			
2	Ccdc8	1.66	1.71	1.79			
3	Wipf1	3.22	4.25	1.26			
4	Emilin1	4.43	4.33	2.29			
5	Zfp105	4.64	3.80	1.21	−1.40		
6	Zmynd15	1.83	1.69	1.54	−1.05	−1.74	−1.90
7	Nptx2	2.30	2.62	1.87	−1.43	−0.60	
8	Sult1c2	1.80	2.43	3.15	−2.98	−0.28	−1.44
9	Npepl1	1.13	2.23	1.78	−1.48	−0.05	0.05
10	Cxcl16	1.39	1.67	1.34	−0.86	−3.51	−2.93
11	Pcdha9	1.27	1.33	1.13	−0.72	−0.88	
12	Col16a1	1.90	1.40	3.24	−0.64	−0.89	−1.15
13	Car13	2.23	1.36	1.27	−0.06	−1.04	
14	Rab19	3.74	3.18	2.02	0.01	−0.76	−1.83
15	Zc3h12d	1.68	1.95	0.87			
16	Tnk1	4.29	4.83	0.66			
17	Prss8	2.05	2.11	0.81			
18	Tgm5	1.68	1.31	0.51	−0.58	−1.15	
19	Cdhr5	4.66	2.35	0.88	−0.88	−0.91	−1.46
20	Pcdhb21	2.04	1.29	0.76	−0.45	−0.73	−1.67
21	Galr3	1.25	1.17	0.61	−0.26	−0.82	−0.27
22	Tanc1	1.09	1.15	0.34			
23	Igfbp4	3.00	2.61	0.36			
24	Ccnb1ip1	3.57	3.00	0.45		−2.20	
25	Akr1c14	1.92	2.19	0.32	−0.82	−1.56	−0.32
26	Cyct	4.45	4.00	−0.28			−0.81
27	Artn	1.74	1.99	−0.12			0.12
28	Ucn3	3.06	2.12	−0.01	−3.17	−1.77	−6.10
29	Crip1	1.19	1.71	−0.13	−2.99	−2.20	−0.67
30	Pcdhb14	1.94	1.50	0.08	−1.97	−0.74	−2.50
31	Gpr142	1.39	2.49	−0.12	−3.57	0.12	−2.97
32	Xndr−trpc2	1.56	1.14	0.16	−1.10	−0.49	−0.16
33	Cldn7	1.32	2.59	−0.06	−1.05	−0.23	−0.24
34	Figf	1.80	1.20	−0.06	−0.77	−1.49	0.06
35	Dennd2d	1.48	1.50	−0.23	−0.77	−0.79	−0.13
36	Pycard	1.02	1.09	−0.05	−0.70	−0.60	0.05
37	Prr22	1.02	1.03	−0.14	−0.52	−0.07	−1.93
38	Spag1	1.29	1.73	−0.06	−0.72	−0.29	−1.06
39	Dmrta2	1.95	1.71	0.14	−0.92	−0.14	
40	Psors1c2	2.63	2.18	−0.01	−0.56	−0.35	−0.48
41	Hvcn1	3.23	2.82	−0.20	−0.46	−1.00	−2.66
42	Tmed6	1.00	1.93	0.16	−0.32	−0.98	−0.16
43	Prom1	1.39	1.40	−0.26	−0.34	−0.99	0.26
44	Tbc1d2	2.59	1.39	−0.23	−0.46	−0.41	0.09
45	Foxj1	1.88	2.20	−0.28	−0.35	−0.02	
46	Siglech	1.51	1.25	0.23	−0.33	−0.23	
47	Ptprn2	1.23	1.13	0.05	−0.40	−0.05	−0.73
48	Plscr4	2.51	2.89	0.04	−0.04	−0.09	−0.57
49	Efcab12	2.02	2.49	−0.01	0.01		
50	Rorb	2.79	1.80	0.01	−0.27		
51	Acot11	1.95	1.79	0.29	−0.29	−1.45	−1.01
52	Cxcl12	1.26	2.38	0.29	−0.29	−2.46	−1.89
53	Dmpk	1.71	1.39	−0.18	−0.22	−1.16	−0.55
54	Cited4	1.84	2.78	−0.07	0.07	−0.94	−0.81
55	Glra1	1.52	1.43	0.12	−0.12	−0.79	−0.72
56	Luzp4	1.15	1.38	−0.11	−0.17	−0.50	−0.73
57	Rasgrf2	1.32	1.90	0.10	−0.10	−0.65	−0.39
58	Pcsk9	1.62	1.02	0.03	−0.03	−0.68	−0.35
59	Amigo3	1.53	1.20	−0.30	0.07	−0.77	−0.07
60	Ptger4	1.73	1.40	−0.27	0.27	−0.33	−0.41
61	Col11a2	2.62	1.85	0.03	−0.28	−0.03	
62	Cldn1	2.61	1.08	−0.16	0.10	−0.10	
63	Med12	1.24	1.62	0.00	−0.09	−0.00	−1.09
64	Esyt2	1.67	1.24	0.18	−0.20	−0.25	−0.18
65	Ncapg2	1.88	1.48	−0.12	0.07	−0.12	−0.08
66	Sh3rf2	1.07	0.61	1.42			
67	Lgals12	2.72	0.67	0.54			
68	Fam196a	1.12	0.57	0.75		−1.66	0.24
69	Prickle3	1.04	0.51	0.70		−0.37	
70	Slc6a19	1.17	0.64	0.57	0.23	−0.65	−0.23

	Annotation	Normalized Data					
	GeneSymbol	H1	H2	H3	ML1	NL2	ML3
71	Uba7	1.80	0.77	0.83	−0.12	−0.80	0.12
72	Agpat2	2.14	0.96	0.32	−3.30	−0.85	−0.32
73	Enpp3	1.94	0.52	0.39	0.16	−0.16	−1.52
74	Vps8	1.49	0.85	−0.11			
75	Egfr	1.19	0.87	0.11	−3.64	−3.04	−0.11
76	Car15	1.04	0.93	0.05	−2.16	−1.81	−0.05
77	Slc8a3	1.08	0.77	−0.07	−1.57	−0.87	−0.14
78	Oacyl	1.20	0.89	−0.26	−1.27	−0.61	−0.19
79	Btnl10	1.35	0.95	−0.27	−1.10	−0.53	−0.24
80	Mtus2	1.13	0.88	−0.23	−1.67	−0.89	−1.00
81	Bricd5	1.57	0.97	0.10	−1.27	−0.63	−0.74
82	Six5	1.14	0.97	−0.18	−1.33	−0.87	0.18
83	Cyp2s1	2.24	0.54	0.17	−4.74	−0.17	−3.84
84	Neurod4	1.64	0.96	0.07	−0.52		
85	Rab43	1.16	0.51	−0.07	−0.71	−0.62	−0.64
86	Chaf1b	1.01	0.76	−0.09	−0.72	−0.81	0.09
87	Cpt1b	1.04	0.81	0.27	−0.66	−0.32	−0.27
88	Pcdhb22	1.47	0.67	0.23	−0.79	−0.23	−1.55
89	Fkbp1b	1.56	0.54	−0.07	−0.52	0.07	−0.91
90	Rhobtb3	1.42	0.93	0.02	−0.92	−0.21	−0.34
91	Pank1	1.00	0.87	0.12	−0.38	−0.52	−0.12
92	Dhcr24	1.12	0.91	−0.03	−0.45	−0.61	0.03
93	Rgag4	1.37	0.66	−0.28	−0.34	−0.32	−1.21
94	Isg15	1.34	0.93	−0.06	−0.42	−0.16	−0.33
95	Tmem37	2.54	0.71	0.13	−0.09		
96	Adhfe1	1.70	0.86	0.24	−0.24		
97	Rnf183	1.76	0.87	−0.06	−0.08		−0.76
98	Abcb4	1.00	0.85	−0.20	−0.30	−1.47	0.14
99	Sap25	1.11	0.92	0.03	−0.24	−0.93	−0.03
100	Ifih1	2.11	0.82	0.28	−0.19	−0.88	0.19
101	Fbln7	1.22	0.35	1.33	−1.94	−1.22	−4.52
102	Fam151a	1.41	0.45	1.54	−2.69	−1.26	−0.12
103	Naip2	1.40	0.35	0.77	−0.30	−3.01	0.30
104	Ak3	1.24	0.47	0.54	−2.11	−4.86	−1.75
105	Usp8	1.13	0.34	0.72	−0.39	−0.82	−0.55
106	Tnfsfm13	1.30	0.40	0.34	−1.80	−3.32	−0.34
107	Slc22a21	1.33	0.42	0.47	−0.51		
108	Prkcd	1.23	0.44	0.44	−1.77	−1.13	−0.85
109	Ripply3	1.10	0.45	0.18	−0.58	−0.36	−0.84
110	Derl3	1.49	0.42	0.20	−1.37	−0.69	−0.20
111	Ap1g2	1.05	0.44	0.02	−0.39	−0.34	−1.03
112	Faah	1.51	0.46	−0.18	−0.90	−0.74	−0.13
113	Ndst4	1.01	−0.15	0.93	−1.65	0.15	−1.53
114	Galnt5	1.69	0.10	−0.10			
115	Chrnb4	1.29	0.27	0.01	−1.09	−0.01	−2.73
116	Zfp219	2.93	0.14	−0.06	−0.58	0.07	
117	Cd44	1.59	−0.05	0.06	−0.41	−1.17	0.05
118	Car2	1.66	0.07	0.20	−0.07	−0.73	−0.73
119	Tlr12	1.40	0.06	−0.16	−0.06	−2.71	−0.71
120	Pter	0.76	1.18	1.38			
121	Il11ra1	0.60	1.39	2.79	−1.13	−0.94	−0.60
122	Gprc5c	0.58	1.10	0.86	−1.37	−0.58	−0.70
123	Tmem200a	0.87	1.65	0.66	−0.66		
124	Apoe	0.63	5.38	0.55	−0.57	−0.66	−0.55
125	Mmd2	0.69	1.33	−0.22	−1.03	−1.28	0.22
126	Six1	0.63	1.06	0.19	−4.33	−1.39	−0.19
127	Asb11	0.91	1.01	0.23	−1.09	−0.47	−0.63
128	Zp2	0.83	1.07	0.04	−1.26	−0.22	0.26
129	Adh1	0.86	1.12	−0.16	−0.93	−0.72	−0.69
130	Man1a	0.90	1.18	−0.08	−0.96	−0.67	0.08
131	Zcwpw2	0.88	1.18	−0.05	−0.57	−0.71	0.05
132	Inmt	0.74	1.71	0.02	−0.59	−0.04	−0.49
133	Dagla	0.91	1.05	0.00	−0.75	−0.00	−0.66
134	Fcrls	0.92	1.11	−0.27	−0.95	−0.13	−0.77
135	Rgs16	0.88	1.58	−0.29	0.10	−1.87	
136	Glt1d1	0.91	1.65	−0.10	−0.29	−0.43	−0.47
137	Fcer1g	0.77	2.92	−0.26	−0.29	−0.04	0.04
138	Ston2	0.55	0.65	0.56		−0.78	−0.26
139	Mppe1	0.67	0.65	0.64	−1.03		−0.34
140	LOC640793	0.65	0.59	0.72	−0.65	−0.59	−0.62

	Annotation	Normalized Data					
	GeneSymbol	H1	H2	H3	ML1	NL2	ML3
141	Rspo4	0.75	0.71	0.80	−0.69	−0.97	−2.52
142	Ffar2	0.92	0.67	0.90	−0.61	−0.23	−1.30
143	Prmt8	0.68	0.83	0.37	−0.15	−0.50	−0.48
144	Dcaf17	0.95	0.68	−0.01			
145	Tmem87a	0.72	0.96	−0.05		−0.53	
146	Hspb1	0.90	0.62	0.01	−1.02	−1.84	−3.54
147	Shf	0.51	0.52	−0.01	−1.39	−1.09	0.01
148	Nabp1	0.83	0.54	0.18	−1.05	−0.73	−0.18
149	Dio1	0.76	0.78	0.12	−1.13	−0.41	−0.11
150	Anks4b	0.76	0.59	0.27	−0.40	−0.20	−1.40
151	Tmem61	0.78	0.82	0.08	−0.91	−0.80	
152	Tfdp2	0.96	0.60	−0.23	−0.72	−0.96	−0.21
153	Plekha2	0.87	0.59	0.29	−0.65	−0.71	−0.17
154	Usp18	0.91	0.72	−0.24	−0.73	−0.38	−1.79
155	Cdkl1	0.69	0.96	0.08	−0.73	−0.01	−0.53
156	Bin3	0.65	0.78	0.10	−0.67	−0.10	−1.18
157	Rhbdl2	0.55	0.76	−0.30	−0.67	−0.26	−1.40
158	Nkd2	0.74	0.78	0.09	−0.34	−0.39	−0.68
159	Kdm2b	0.96	0.57	−0.15	−0.35	−0.28	−1.29
160	Lrrc16b	0.50	0.52	−0.02	−0.37	−0.11	−1.94
161	Tnfsf12	0.82	0.77	−0.00	0.00	−1.61	−0.13
162	Tmem8	0.75	0.64	−0.16	−0.25	−1.81	0.08
163	Myo1e	0.96	0.52	0.19	−0.27	−0.99	−0.53
164	Ubxn2b	0.77	0.56	0.20	−0.27	−0.59	−0.96
165	Gem	0.84	0.62	0.12	−0.12	−0.56	−0.90
166	Kidins220	0.76	0.70	0.27	−0.20	−0.65	−0.58
167	Flnb	0.54	0.66	0.02	−0.28	−0.36	−1.36
168	Fasn	0.87	0.62	−0.29	−0.08	−0.45	−1.34
169	Cacna1d	0.73	0.88	−0.08	−0.17	−0.03	−1.59
170	Zhx2	0.57	0.42	1.00	−0.46	−0.27	
171	Adcy7	0.85	0.32	2.00	0.26	−1.70	−0.26
172	Mtap7d3	0.75	0.43	0.39	−2.23	−1.61	−0.39
173	Gbe1	0.76	0.43	0.28			
174	Eva1c	0.62	0.33	0.22	−1.77	−1.07	0.28
175	Ncor1	0.64	0.34	0.03	−1.45	−0.56	−0.33
176	Tagln2	0.58	0.46	−0.04	−2.69	−0.43	−0.57
177	Nnt	0.73	0.34	0.05	−1.79	−0.05	−1.01
178	Rcbtb1	0.75	0.40	−0.30	−0.78	−0.84	
179	Fbxl17	0.90	0.39	−0.03	−0.88	−0.64	−0.35
180	Cdhr1	0.73	0.44	0.03	−0.76	−0.24	−2.87
181	Crybb1	0.73	0.44	0.06	−0.65	−0.21	−3.05
182	Dph3	0.56	0.33	−0.04	−0.59	0.04	−1.68
183	Mfsd1	0.79	0.47	0.24	−0.46	−1.96	0.07
184	Psd	0.51	0.40	−0.18	−0.42	0.12	−2.11
185	Itpkb	0.57	0.32	0.14	−0.14	−0.48	−2.47
186	Il20rb	0.83	0.45	−0.04	0.04	−1.04	−2.41
187	Fam149b	0.79	0.37	−0.12	0.06	−0.06	−2.08
188	Abcc5	0.86	0.33	0.05	−0.05	−0.11	−1.75
189	Kbtbd11	0.92	0.23	1.55	−1.23	−1.31	0.24
190	Xlr	0.69	0.03	0.70	−6.15	−4.17	−5.32
191	Tenm4	0.61	0.18	0.56	−1.48	−0.18	−0.49
192	Vmn1r65	0.59	−0.07	0.76	−0.94	0.06	−1.42
193	Slx	0.63	−0.06	0.25	−6.63	−4.29	−5.09
194	Mrgpra2b	0.67	0.13	−0.13	−1.59	−1.03	0.28
195	Cacna1c	0.60	−0.02	0.05	−0.91	−0.83	−0.86
196	Pnma2	0.56	0.16	0.18	−0.63	−0.16	−2.00
197	Ddx31	0.85	0.17	0.17	−0.98	−0.12	−0.82
198	Rsph3a	0.75	0.09	−0.09	−0.40	−0.61	−1.70
199	Dennd4c	0.89	0.24	0.23	−0.34	−0.41	−0.98
200	Sytl4	0.87	0.04	−0.20	−0.03	−0.74	−2.00
201	Lefty1	0.34	1.01	0.69	−0.69		−3.55
202	Sphk1	0.39	2.48	0.05	−1.74	−2.62	−0.05
203	Glis1	0.44	2.92	0.13	−0.77	−1.06	−0.13
204	Adamtsl2	0.44	0.75	0.50		−1.68	−0.44
205	Mybph	0.48	0.54	0.57	−2.09	−0.90	−0.48
206	Crp	0.49	0.93	0.43	−1.18	−0.51	−0.24
207	Rnase4	0.47	0.73	0.44	−0.44	−0.64	−1.18
208	Degs2	0.45	0.63	−0.09	−1.20	−1.48	−0.27
209	Pou5f2	0.39	0.96	0.14	−1.44	−0.75	−0.47
210	Slain1	0.41	0.53	0.07	−0.62	−0.07	−1.42

	Annotation	Normalized Data					
	GeneSymbol	H1	H2	H3	ML1	NL2	ML3
211	Cep192	0.35	0.69	0.20	−0.77	−0.56	−0.80
212	S100pbp	0.39	0.68	0.08	−0.93	−1.27	−0.52
213	Phyhip	0.31	0.84	0.27	−0.27	−0.41	−2.75
214	Inhba	0.37	0.31	1.02	−0.31	−1.26	−1.74
215	Kcnb1	0.33	0.36	0.34	−1.09	−0.83	−0.18
216	Adora1	0.41	0.39	−0.22	−1.00	−2.27	0.22
217	Fbxo17	0.44	0.50	0.05	−0.05	−1.07	−2.16
218	Arap1	0.46	0.35	0.05	−0.05	−2.17	−0.38
219	Ugt3a2	0.42	0.14	2.26		−0.35	−0.14
220	Acot6	0.31	0.28	1.17	−0.25	0.25	−1.25
221	Vps37c	0.48	0.07	0.43	−1.06	−0.89	−1.32
222	Dclre1c	0.40	0.24	0.02	−1.02	−1.30	−0.02
223	Car5b	0.46	−0.05	0.05	−1.01	−1.53	−2.56
224	Scnn1b	0.37	0.11	−0.11	−1.16	−1.02	−1.21
225	Nxph1	0.34	0.10	−0.10	−1.96	−1.17	0.10
226	Hexim1	0.35	0.23	−0.01	−0.70	−0.19	−1.70
227	Necab2	0.02	1.48	1.46	−0.02	−1.74	−1.40
228	Dapl1	0.01	1.72	0.12	−1.98	−5.28	−0.01
229	Llgl2	0.19	1.00	−0.19	−1.15	−0.89	−0.20
230	Foxc1	0.22	1.22	−0.00	−1.61	0.00	−0.64
231	Shd	0.10	1.29	0.13	0.17	−1.37	−1.86
232	Agt	0.01	0.86	1.69	−1.42	−0.44	−0.71
233	LOC10004170ε	−0.13	0.61	3.37	0.10	−0.37	−0.10
234	Fam117a	−0.12	0.57	0.55	−1.88	−0.71	−1.61
235	Defb1	0.30	0.52	0.73	−2.01	−0.84	−0.30
236	Lyve1	0.08	0.82	0.33	−1.48	−0.60	−2.88
237	Dhdh	−0.24	0.73	0.24	−3.71	−1.36	−5.01
238	Fbxo32	0.20	0.60	0.25	−1.31	−0.84	−0.12
239	Acsbg1	0.26	0.82	−0.14	−1.55	−0.63	−1.28
240	Syn2	0.14	0.70	−0.14	−0.88	−0.83	−1.66
241	Dscam	0.09	0.83	−0.09	−0.31	−1.23	−1.50
242	Tspan15	0.03	0.43	0.29	−1.85	−1.10	−0.03
243	Igf1r	0.09	0.43	−0.06	−2.35	−1.30	−0.23
244	Myo18a	0.27	0.31	−0.06	−0.77	−1.07	−1.06
245	Esyt1	0.12	0.32	0.06	−0.54	−0.50	−1.64
246	Cd40	0.10	0.21	1.31	−1.40	−1.26	−0.10
247	Rab33a	−0.28	0.28	2.47	−1.30	0.29	−0.89
248	St6galnac5	0.27	0.09	0.51	−1.12	−0.62	−0.89
249	Fam163b	0.11	0.04	0.98	−2.34	−0.82	−0.04
250	Chmp4b	0.03	0.19	0.61	−0.83	−0.81	−0.54
251	Tacr3	0.28	0.07	0.85	−0.07	−1.48	−0.93
252	Zfp647	0.21	−0.05	0.40	0.05	−4.15	−0.44
253	Inppl1	0.09	0.17	0.03	−1.34	−1.13	−0.66
254	Pax4	0.28	0.27	−0.11	−2.25	−0.75	−3.32
255	Pcdhb16	0.01	−0.01	0.25	−1.25	−0.37	−1.50
256	Rnf135	0.10	0.11	0.15	−0.68	−1.11	−1.43
257	Slitrk6	0.01	0.11	0.10	−0.73	−0.01	−2.11
258	Kcnmb2	0.18	0.20	−0.17	−0.85	−0.15	−1.87
259	Rec8	−0.01	0.11	0.01	−0.30	−0.17	−5.19
260	Prrt1	0.30	0.13	0.08	−0.08	−5.06	−0.53
261	Osbpl10	0.16	0.26	0.03	−0.20	−1.10	−1.29
262	Pard3	0.23	0.12	0.29	−0.28	−0.12	−2.18

・・・1 ＜ Normalized Data
・・・0.5 ＜ Normalized Data ≦ 1
・・・0.3 ＜ Normalized Data ≦ 0.5
・・・-0.3 ＜ Normalized Data ≦ 0.3
・・・-0.5 ＜ Normalized Data ≦ -0.3
・・・-1 ＜ Normalized Data ≦ -0.5
・・・Normalized Data ≦ -1
・・・ Not detected or compromised

List of genes preferentially expressed in sublines with mildly low glucose responsiveness selected by our criteria described in the text

	Annotation	Normalized Data					
	GeneSymbol	H1	H2	H3	ML1	ML2	ML3
1	Fam126a				6.09	5.39	4.64
2	Tfpi				4.51	1.80	7.78
3	Eya2				3.15	1.48	1.23
4	Cmya5				3.09	1.93	3.41
5	Sec14l3				2.32	3.12	1.21
6	Mgll				2.08	1.11	3.32
7	Esx1				2.46	1.75	2.70
8	H2-Eb1				1.82	3.44	3.44
9	Mylk				1.75	1.08	3.12
10	Pik3cd				1.33	1.12	1.74
11	Afm				1.11	1.06	2.59
12	Itga1				1.90	1.10	0.89
13	Maats1				1.89	1.56	0.74
14	Tpsg1				4.19	0.97	1.74
15	C4bp				3.86	0.51	6.84
16	Morc1				2.61	0.88	2.63
17	Lrmp				2.36	0.50	2.26
18	Mgst2				1.67	0.99	1.46
19	Olfr1383				1.52	0.41	1.89
20	Robo3				1.50	0.42	2.88
21	Stat4				1.13	0.32	0.32
22	Sowaha				0.88	1.45	2.25
23	Gys2				0.61	1.38	1.62
24	Slc8a1= Ncx1				0.94	1.10	0.09
25	Ccdc150				0.89	0.57	1.23
26	Mamdc2				0.84	0.72	1.08
27	P4ha3				0.69	0.50	1.60
28	Tubb1				0.63	0.52	1.17
29	Slc37a2				0.85	0.38	0.36
30	Pigb				0.59	0.45	0.10
31	Fam228b				0.97	0.15	0.86
32	Dpys				0.43	1.31	0.97
33	Ankrd45				0.41	0.62	0.77
34	Kif7				0.31	0.77	0.82
35	Gckr				0.48	0.49	1.36
36	Rnasel				0.40	0.43	1.31
37	Mogat1				0.43	0.08	3.33
38	Cbln1				0.31	0.19	0.84
39	Pbx1				0.26	0.34	0.93
40	Adcy5			−1.25	0.15	1.82	−0.15
41	Foxa1			−3.42	0.29	−0.29	1.95
42	Sox11			−0.72	1.69	0.68	3.69
43	Pik3c2b			−0.89	1.18	0.02	−0.02
44	Vsig1			−0.41	1.85	1.91	0.59
45	Pcdh8			−0.02	2.04	3.18	2.22
46	Col14a1			0.11	1.76	2.16	2.27
47	P2ry14			0.04	1.55	1.18	1.46
48	Dpysl3			0.02	1.80	1.68	−0.02
49	Dmtn			−0.15	4.44	0.72	0.15
50	Plin3			0.13	2.70	0.42	2.00
51	Klhl30			−0.13	1.56	0.23	2.93
52	Syt17			0.25	1.37	0.08	2.40
53	Ngef			−0.09	0.99	1.31	1.59
54	Atp6ap1l			0.02	0.51	2.22	0.33
55	Enkur			−0.16	0.60	0.92	1.45
56	Prtn3			−0.14	0.37	0.82	1.48
57	Nrg4			−0.08	0.08	1.69	2.85
58	Slc32a1			−0.15	0.15	1.97	5.25
59	Rab3c			−0.07	0.07	1.59	1.52
60	Fbxo41		−1.92	−1.59	−0.09	0.44	0.40
61	Camk1d		−1.67	−0.87	0.87	1.14	1.51
62	Fhl2		−1.12	−0.32	3.39	0.32	4.02
63	Dclk2		−1.08	−0.42	2.53	0.03	1.08
64	Nek11		−1.14	−0.44	0.67	0.86	0.44
65	Vmn2r96		−1.57	−0.38	0.38	1.70	1.28
66	Rasl10a		−1.84	0.23	1.81	0.89	3.19
67	Podxl2		−2.00	−0.29	1.28	0.98	0.29
68	Lingo3		−1.52	0.06	1.33	0.80	−0.06
69	Anxa10		−1.18	0.30	2.76	0.33	−0.30
70	Reck		−1.01	−0.04	1.07	0.04	0.97

	Annotation	Normalized Data					
	GeneSymbol	H1	H2	H3	ML1	NL2	ML3
71	Hs6st2		−1.19	0.07	2.49	−0.07	2.81
72	Galnt16		−1.14	−0.13	0.91	0.19	0.92
73	Alg13		−3.05	0.10	0.39	0.42	0.24
74	Plp1		−2.99	0.30	0.15	0.49	0.18
75	Tox		−1.10	−0.14	0.14	0.38	0.92
76	Ak8		−1.81	−0.10	0.10	0.21	1.44
77	Bpifb4		−0.91	−0.37	1.16	1.19	0.11
78	Syne1		−0.52	−0.36	3.14	0.97	0.36
79	Arhgap22		−0.79	−0.31	4.11	−0.00	0.81
80	Pthlh		−0.95	−0.25	0.93	−0.03	1.69
81	Ube3a		−0.96	−0.11	0.43	0.58	0.92
82	Zfp467		−0.93	−0.17	0.33	0.17	2.08
83	Wdr16		−0.48	−0.17	1.26	1.52	2.89
84	Cers3		−0.31	−0.01	1.17	0.48	1.11
85	Dmkn		−0.45	−0.01	0.39	0.01	2.60
86	Hrasls		0.21		0.71	−0.21	3.44
87	Pcsk6		0.07	−2.57	2.00	2.36	1.02
88	Sntg2		0.26	−1.66	−0.16	2.83	3.02
89	Fam171a2		−0.16	−1.28	0.16	0.44	0.58
90	Shisa7		0.08	−1.22	−0.08	0.18	1.36
91	Itgb7		−0.18	−0.68	1.59	0.36	0.28
92	Trim12c		0.06	−0.51	0.49	−0.06	2.77
93	Cd276		−0.02	−0.34	1.05	0.02	1.59
94	Evc		0.20	−0.20	3.93	2.86	2.62
95	St6gal1		0.28	−0.28	1.79	1.77	0.83
96	Fam107b		−0.17	0.17	3.60	0.59	1.94
97	Mcam		0.01	−0.06	1.18	−0.25	2.40
98	Gsta3		−0.06	−0.07	0.17	1.11	2.20
99	Rftn1	−1.90		−0.64	1.90	1.81	0.64
100	Xdh	−1.61		0.26	1.20	0.23	0.43
101	Six3	−1.24		0.02	−0.28	1.32	1.54
102	Uchl1	−4.12	−3.85	−1.56	2.07	1.56	2.87
103	Kcnj12	−3.41	−1.51	−1.01	1.62	1.01	1.73
104	Dbn1	−1.52	−1.24	−0.73	1.55	1.08	0.39
105	Nrxn2	−1.98	−2.02	−0.51	2.05	0.51	0.78
106	Smarcd3	−2.30	−1.56	−0.66	1.32	0.05	0.77
107	Vstm2b	−1.26	−1.31	−0.51	1.09	−0.28	1.65
108	Kcnc1	−1.54	−1.71	−0.68	0.88	0.68	1.37
109	Lrrc4b	−1.28	−1.15	−0.86	0.21	1.13	0.23
110	Hck	−2.05	−1.89	−0.32	2.12	1.73	0.69
111	Stmn4	−2.01	−3.15	−0.43	2.40	0.43	2.33
112	Lin28b	−4.94	−1.62	−0.32	0.82	0.58	0.32
113	Nr2f1	−1.70	−2.91	0.09	2.30	4.19	4.78
114	Eps8l1	−1.36	−1.32	0.28	1.07	1.63	0.45
115	Edil3	−1.09	−1.78	0.20	1.05	2.10	0.46
116	Sec14l2	−2.24	−1.31	0.22	1.37	0.89	1.68
117	Nefh	−1.37	−1.33	0.18	1.28	0.65	−0.18
118	Fam49a	−1.16	−1.71	0.19	1.08	0.09	1.07
119	Tcn2	−2.16	−1.68	0.16	1.07	−0.16	1.18
120	Gng3	−2.07	−1.50	0.23	1.42	−0.23	0.35
121	Slc1a2	−1.22	−2.21	0.24	1.06	−0.06	0.06
122	Smtn	−1.39	−1.35	0.23	1.65	0.16	−0.16
123	Inpp5j	−1.14	−1.24	0.17	1.49	0.01	−0.01
124	Lrrtm2	−1.45	−2.04	−0.20	0.59	1.41	0.20
125	Nipsnap1	−1.53	−1.22	0.04	0.88	0.59	0.61
126	Radil	−1.51	−1.49	0.23	0.82	0.61	0.23
127	Slit1	−2.03	−1.46	−0.06	0.57	0.42	1.13
128	Akap17b	−1.27	−2.43	−0.13	0.97	0.44	0.88
129	Oas1d	−1.12	−1.44	0.29	0.85	0.47	0.80
130	Atxn7l1	−1.02	−1.08	0.03	0.88	0.36	0.36
131	Rab9b	−4.16	−2.70	0.17	0.90	0.49	−0.17
132	Thoc5	−1.56	−1.41	0.20	0.83	0.30	−0.17
133	Yjefn3	−1.51	−1.39	−0.18	0.66	0.27	0.52
134	Trp53i11	−1.63	−1.36	0.06	0.78	−0.06	0.88
135	Slc45a1	−4.53	−1.45	−0.07	0.53	0.07	0.74
136	Gas2l1	−1.48	−1.49	0.25	0.56	−0.12	0.12
137	Slc41a3	−4.05	−2.88	0.08	0.65	−0.08	0.10
138	Arrb1	−2.21	−1.84	0.01	0.66	−0.30	−0.01
139	Dusp18	−1.50	−1.57	0.28	0.55	0.13	−0.13
140	Sf3a1	−1.61	−1.22	0.18	0.89	0.14	−0.14

	Annotation	Normalized Data					
	GeneSymbol	H1	H2	H3	ML1	NL2	ML3
141	Ncan	−2.10	−1.47	0.10	0.41	0.93	−0.10
142	Pes1	−1.54	−1.44	0.16	0.47	0.39	−0.16
143	Zcchc18	−4.18	−2.32	0.20	0.31	0.49	0.24
144	Cacna1g	−1.52	−1.58	0.24	0.37	0.44	−0.24
145	Ap1b1	−1.30	−1.23	0.09	0.39	0.34	−0.09
146	Zfyve28	−1.71	−1.52	−0.12	0.49	0.09	0.96
147	Popdc3	−1.03	−1.15	−0.07	0.46	0.07	0.56
148	Uqcr10	−1.39	−1.17	0.11	0.37	0.23	0.21
149	Mtmr3	−1.83	−1.35	0.29	0.39	0.15	−0.15
150	Drg1	−1.90	−1.38	0.04	0.48	0.26	−0.04
151	Emid1	−3.00	−2.64	−0.02	0.02	1.17	2.95
152	Mkrn3	−2.82	−2.30	0.25	−0.25	1.02	0.35
153	Slc35f3	−3.53	−1.21	0.15	0.17	1.34	−0.15
154	Mapt	−1.06	−1.28	−0.16	0.16	0.63	0.86
155	Olfr1466	−3.33	−3.27	0.29	−0.17	0.67	0.17
156	Clec11a	−1.65	−1.03	−0.00	0.00	0.48	1.04
157	Mum1l1	−5.54	−3.08	0.03	0.02	0.47	−0.02
158	Rab30	−1.24	−1.03	0.26	0.27	−0.26	1.02
159	Neto1	−2.06	−1.76	0.01	0.18	−0.01	0.82
160	Nrg1	−4.10	−2.87	0.29	0.06	−0.06	0.17
161	Txnrd3	−1.19	−1.72	0.05	−0.05	0.09	0.28
162	Prex1	−2.07	−1.58	0.09	−0.00	0.06	0.00
163	Rfxank	−1.63	−1.39	0.09	0.18	−0.09	0.10
164	Igsf1	−1.51	−0.88	−0.59	1.18	0.46	1.34
165	Smtnl2	−2.92	−0.55	−0.46	0.59	1.29	3.43
166	Sult4a1	−2.66	−0.61	−0.31	0.90	0.38	0.31
167	Cpne5	−1.24	−0.76	−0.03	1.65	0.99	−0.11
168	Garnl3	−1.26	−0.61	−0.04	1.36	0.33	1.46
169	Slc38a9	−1.78	−0.76	0.17	1.06	0.49	−0.17
170	Pcdhb2	−1.06	−0.69	−0.05	1.21	0.44	−0.12
171	Arrdc4	−1.63	−0.91	0.23	1.07	−0.21	2.03
172	Mtfp1	−1.26	−0.81	−0.04	0.97	1.06	0.04
173	Kdelr3	−2.33	−0.51	−0.15	0.72	0.65	0.46
174	Zfp454	−1.25	−0.66	−0.08	0.94	0.46	0.62
175	Ccnj	−1.04	−0.64	−0.01	0.52	0.41	0.64
176	Clstn3	−1.14	−0.66	0.24	0.91	0.31	0.41
177	Shank2	−1.25	−0.96	0.16	0.94	−0.16	1.42
178	Mmp17	−1.26	−0.75	−0.08	0.51	0.08	0.54
179	Adap1	−1.39	−0.74	−0.20	0.64	0.16	0.45
180	Iqsec1	−1.50	−0.69	−0.01	0.62	0.29	0.01
181	Plxnd1	−1.68	−0.75	0.22	0.47	−0.22	2.46
182	Matn2	−1.71	−0.95	0.15	0.48	−0.08	1.13
183	Tln2	−1.08	−0.76	−0.16	0.38	0.16	1.11
184	Dlgap4	−1.23	−0.95	−0.12	0.33	0.12	0.66
185	Sfxn5	−3.23	−0.95	0.15	0.43	−0.15	0.43
186	Zfp385a	−1.21	−0.98	−0.18	0.47	0.28	0.18
187	Itga4	−1.78	−0.95	0.15	−0.15	0.72	0.20
188	Spag4	−1.08	−0.82	−0.23	0.27	0.23	1.48
189	Nme5	−1.08	−0.82	−0.17	0.30	0.17	0.65
190	Lonrf1	−3.46	−0.82	0.22	0.15	−0.11	0.82
191	Egln3	−2.00	−0.72	0.13	−0.09	0.09	0.67
192	Ptprt	−2.24	−0.30	−0.77	1.58	0.86	0.30
193	Pde4a	−1.51	−0.35	−0.51	0.40	0.22	0.28
194	Prickle1	−1.81	−0.45	0.30	2.88	0.85	2.22
195	Akap6	−1.34	−0.49	0.05	1.68	0.29	−0.05
196	Cdh4	−1.20	−0.32	0.07	1.08	−0.07	1.07
197	Smim18	−1.28	−0.47	−0.09	0.71	0.41	0.09
198	Pcdhb4	−1.81	−0.36	−0.08	0.77	0.13	0.72
199	Dtx1	−1.75	−0.49	0.01	0.35	1.02	0.72
200	Fam78a	−1.26	−0.39	0.23	0.01	−0.01	1.66
201	Plekhd1	−1.98	0.14	−1.34	0.33	0.18	−0.14
202	Wnt4	−2.50	−0.05	−1.82	0.41	0.05	0.06
203	Arhgef6	−2.64	−0.09	−1.02	0.21	0.52	0.09
204	Slc25a13	−1.30	0.22	−2.32	−0.01	1.05	2.70
205	Sh3bgr	−1.42	0.18	−0.84	1.08	−0.18	1.11
206	Aloxe3	−1.67	0.00	−0.67	0.63	0.25	−0.00
207	Plb1	−1.23	−0.12	−0.44	0.51	0.12	1.03
208	Plin5	−2.13	−0.28	0.28	2.19	1.04	2.31
209	Car12	−1.17	0.07	−0.07	1.34	0.31	1.06
210	Lgi2	−2.06	−0.27	−0.03	1.01	0.03	1.20

	Annotation	Normalized Data					
	GeneSymbol	H1	H2	H3	ML1	NL2	ML3
211	Plcg2	−1.63	0.11	−0.11	2.45	0.20	0.83
212	Prkcb	−3.13	0.16	0.22	1.19	−0.04	0.04
213	Vegfc	−1.22	−0.28	0.18	0.57	0.39	1.77
214	Car11	−1.13	0.20	−0.25	−0.20	1.39	0.93
215	Spry1	−1.23	0.13	−0.13	−0.26	−0.27	2.41
216	Sept9	−2.58	−0.04	0.09	0.07	0.04	0.41
217	Hecw2	−0.98		−1.12	1.14	0.32	1.90
218	Gpr1	−0.64	−1.42	−1.85	1.02	0.24	0.77
219	Myo18b	−0.51	−1.89	−0.41	0.94	0.41	1.22
220	Kdm4d	−0.54	−1.07	0.11	1.07	0.66	0.61
221	Lix1	−0.54	−1.23	0.25	2.18	−0.11	1.18
222	Mthfr	−0.91	−1.01	−0.05	0.86	0.05	0.18
223	Ttll11	−0.75	−1.15	−0.03	0.42	1.02	−0.01
224	Tex26	−0.93	−1.19	−0.10	0.42	0.10	0.66
225	Cdkn1c	−0.92	−1.27	0.24	0.14	1.11	−0.11
226	Tmem116	−0.82	−1.12	−0.27	0.27	0.61	1.68
227	Agpat4	−0.76	−1.04	−0.09	0.13	0.31	0.71
228	Tpm1	−0.91	−0.52	−0.62	1.63	0.74	0.35
229	Ccdc112	−0.55	−0.61	−0.60	0.97	0.43	0.95
230	Atp9a	−0.77	−0.95	−0.68	0.80	0.59	0.18
231	Zfp365	−0.53	−0.75	−0.65	0.60	0.00	0.78
232	Pde6c	−0.64	−0.56	−0.68	0.36	0.28	0.77
233	Dab1	−0.74	−0.63	−0.48	1.78	1.41	1.07
234	Tspan2	−0.57	−0.58	−0.42	0.60	0.18	0.75
235	Rnd3	−0.74	−0.55	−0.37	1.00	0.99	0.88
236	Fam78b	−0.57	−0.70	−0.38	0.63	0.38	0.68
237	Tyro3	−0.50	−0.75	−0.47	0.46	1.05	1.47
238	Pcdhb5	−0.82	−0.54	−0.10	1.04	0.90	0.44
239	Scn1a	−0.97	−0.78	0.02	1.92	0.77	0.04
240	Mttp	−0.81	−0.59	−0.18	1.03	0.09	1.01
241	Hs2st1	−0.87	−0.72	0.29	0.82	0.72	0.75
242	Park2	−0.88	−0.61	0.19	0.73	0.75	0.62
243	Nelfcd	−0.79	−0.56	0.04	0.73	0.64	0.63
244	Pcdhb3	−1.00	−0.54	−0.13	0.78	0.40	0.44
245	Fbxo15	−0.95	−0.74	−0.08	0.72	0.08	1.38
246	Zfp354b	−0.91	−0.71	−0.01	0.68	0.05	0.64
247	Rwdd3	−0.91	−0.79	−0.16	0.99	0.16	0.34
248	Steap3	−0.98	−0.52	−0.23	0.91	0.17	0.33
249	Nyap2	−0.64	−0.64	−0.24	0.31	1.03	0.38
250	Tmem53	−0.99	−0.57	−0.21	0.32	0.58	1.30
251	Ppp1r32	−0.92	−0.73	0.06	0.46	0.16	1.03
252	Adc	−0.74	−0.79	−0.16	0.07	0.23	1.14
253	Tmem74b	−0.83	−0.50	−0.26	0.26	0.55	0.73
254	Zfp955b	−0.50	−0.34	−1.12	0.52	0.29	0.23
255	Hist1h1a	−0.66	−0.35	−0.59	0.35	1.29	0.51
256	Mex3a	−0.82	−0.46	−0.34	0.08	0.09	1.40
257	Slc18b1	−0.61	−0.32	−0.24	1.01	0.64	0.53
258	Vax2	−0.53	−0.49	−0.28	1.18	1.00	0.40
259	Asic5	−0.67	−0.49	0.22	0.92	1.94	0.05
260	Neurl2	−0.81	−0.33	0.02	0.52	0.10	1.92
261	Sgtb	−0.71	−0.45	−0.07	0.46	1.29	0.07
262	Asns	−0.98	−0.42	−0.20	0.09	0.22	1.28
263	Mfap5	−0.80	−0.08	−1.06	1.56	0.22	0.08
264	Epdr1	−0.94	0.10	−2.14	1.01	−0.10	0.24
265	Gast	−0.86	−0.24	−1.02	0.56	0.92	0.47
266	Nyap1	−0.53	−0.29	−1.62	0.89	0.57	0.44
267	P2rx3	−0.73	−0.15	−1.35	0.70	0.18	0.16
268	Stk32a	−0.80	−0.11	−1.14	0.03	0.43	0.90
269	Svop	−0.64	0.19	−0.68	1.45	1.81	0.93
270	Smox	−0.76	0.00	−0.97	0.63	−0.11	1.55
271	Fank1	−0.54	−0.09	−0.61	0.20	0.22	1.84
272	Epha8	−0.98	−0.21	−0.42	1.18	0.32	0.21
273	Aldh1b1	−0.74	0.07	−0.38	0.97	−0.07	2.05
274	Mgst3	−0.94	−0.10	−0.47	0.15	−0.01	1.51
275	Sorcs2	−0.64	−0.09	−0.06	1.80	2.63	0.06
276	Mfhas1	−0.70	−0.19	0.19	1.35	0.73	0.70
277	Folr1	−0.63	−0.15	−0.14	2.21	0.41	0.14
278	Dusp22	−0.61	−0.19	−0.03	1.63	0.03	1.81
279	Atp1a3	−0.79	−0.25	0.06	1.45	0.11	1.03
280	Pcdhb7	−0.53	−0.02	0.19	0.74	−0.04	2.12

	Annotation	Normalized Data					
	GeneSymbol	H1	H2	H3	ML1	NL2	ML3
281	Kiss1r	−0.88	−0.18	−0.16	0.83	0.14	0.95
282	Nxnl2	−0.60	−0.05	0.05	0.42	0.50	2.05
283	Lrp2bp	−0.80	−0.16	−0.00	0.00	0.68	1.59
284	Etv4	−0.81	−0.22	−0.05	0.05	0.53	1.39
285	Adcy2	−0.50	−1.20	−1.50	0.27	0.35	0.32
286	Jag1	−0.47	−1.78	−0.08	0.41	0.55	0.25
287	Cep85l	−0.38	−1.50	0.03	0.35	0.46	0.70
288	Dusp8	−0.39	−1.21	0.01	0.61	−0.01	1.12
289	Arid3a	−0.44	−0.51	−1.25	0.47	0.54	0.73
290	Hlx	−0.46	−0.57	−0.60	0.66	−0.10	1.62
291	Pak7	−0.33	−0.70	−0.85	0.53	0.95	0.01
292	Slc7a3	−0.30	−0.61	−0.30	0.70	0.08	1.51
293	Cd300lf	−0.45	−0.54	−0.42	0.96	0.94	1.34
294	Adam23	−0.30	−0.63	−0.12	0.51	0.12	1.53
295	Porcn	−0.37	−0.72	−0.13	0.26	0.29	1.33
296	Slc39a11	−0.45	−0.51	−0.28	1.10	0.41	0.61
297	Nsg1	−0.37	−0.46	−0.61	0.76	0.80	0.27
298	Tnr	−0.39	−0.35	−0.79	0.35	0.81	1.25
299	Zfp951	−0.46	−0.38	−0.36	1.18	0.79	0.32
300	Slc1a6	−0.41	−0.47	0.28	2.78	0.06	2.97
301	Sytl3	−0.36	−0.44	−0.04	0.04	1.17	1.62
302	Pabpc1l	−0.44	−0.12	−1.94	1.43	0.44	2.31
303	Celf3	−0.34	−0.17	−1.07	0.64	0.47	0.34
304	Slc5a10	−0.41	−0.09	−0.58	1.65	2.19	0.70
305	Akap12	−0.36	−0.07	−0.62	2.83	0.07	2.10
306	Tmeff1	−0.37	−0.03	−0.91	0.74	0.20	1.24
307	Kcna4	−0.45	−0.17	−0.41	1.04	0.91	0.14
308	Kalrn	−0.35	−0.28	−0.35	0.63	0.78	1.11
309	Pde8b	−0.39	−0.18	0.06	0.90	1.81	−0.08
310	Ctsh	−0.30	−0.13	−0.05	0.65	0.32	3.85
311	Gpr88	0.20		−1.17	0.57	−0.26	2.08
312	Nrp2	−0.07		−0.29	0.68	0.07	1.88
313	Kcnc4	0.17	−1.17	−1.08	1.28	0.35	1.31
314	Srgap1	−0.17	−1.10	−1.11	0.48	0.18	0.66
315	Vil1	−0.25	−1.47	−0.99	0.28	0.60	2.10
316	Ccnjl	−0.14	−1.27	0.05	1.46	1.56	1.03
317	Speg	0.12	−1.11	−0.12	0.80	1.44	0.26
318	L3mbtl3	−0.12	−1.44	0.17	0.55	0.12	1.14
319	Sept6	−0.30	−1.09	−0.11	0.39	0.63	1.48
320	Lats2	0.20	−2.56	−0.20	0.43	0.60	0.82
321	Skor1	−0.05	−1.13	−0.24	0.05	0.77	1.51
322	Frem1	−0.22	−0.64		0.98	0.88	0.22
323	Zpld1	−0.14	−0.86	−1.26	0.42	0.29	2.01
324	Ccny	−0.24	−0.80	−1.49	0.16	0.53	0.17

	Annotation	Normalized Data					
	GeneSymbol	H1	H2	H3	ML1	NL2	ML3
325	Tspan18	−0.27	−0.78	−0.90	1.37	0.30	0.59
326	Tnnt1	0.03	−0.55	−0.70	1.00	0.75	1.32
327	Shisa4	−0.13	−0.59	−0.46	1.06	0.22	1.60
328	Cpne2	−0.20	−0.76	−0.06	1.22	0.45	0.81
329	Sema6b	−0.17	−0.94	−0.13	1.32	0.44	0.47
330	Lhfpl2	0.29	−1.00	−0.29	0.76	1.27	1.01
331	Calb1	−0.10	−0.61	0.10	0.37	0.39	1.89
332	Flrt1	0.13	−0.75	−0.19	0.41	−0.13	2.65
333	Gnas	−0.10	−0.36	−2.11	1.00	0.10	−0.24
334	Hes5	0.03	−0.30	−1.37	0.30	0.07	0.99
335	Limch1	−0.16	−0.40	−1.03	0.22	0.42	1.35
336	Ccrn4l	−0.12	−0.43	−0.85	1.20	−0.04	0.54
337	Ctf2	−0.26	−0.49	−0.13	1.27	0.96	0.13
338	Mmp24	−0.19	−0.49	−0.19	0.54	1.03	1.30
339	Macrod2	−0.21	−0.36	−0.29	0.95	1.64	0.74
340	Smc2os	−0.26	−0.47	−0.16	0.59	0.64	1.09
341	Sort1	−0.26	−0.43	−0.23	0.45	0.13	1.54
342	Hhip	−0.19	−0.18	−1.51	1.19	1.76	3.14
343	Enc1	0.02	−0.02	−1.65	0.83	0.97	0.32
344	Hmgb1	−0.15	−0.19	−1.46	0.82	0.72	−0.28
345	Lypd6	−0.03	−0.19	−1.19	0.87	0.03	1.74
346	Fgf12	0.02	−0.04	−1.01	0.48	0.72	1.03
347	Ccdc113	−0.04	−0.23	−0.89	1.51	1.46	0.04
348	Tmem171	0.22	−0.22	−0.96	2.39	0.70	3.05
349	Tmcc3	−0.20	−0.25	−0.91	1.22	0.80	2.62
350	Nipal2	0.09	−0.09	−0.97	1.58	−0.09	0.75
351	Txnip	0.00	−0.00	−0.58	2.81	0.18	0.79
352	Poln	0.22	0.12	−0.75	0.72	−0.14	2.22
353	Ctf1	−0.08	−0.11	−0.75	0.85	0.08	1.87
354	Coro1a	0.07	−0.17	−0.41	1.45	0.68	0.98
355	Tspan33	−0.08	0.08	−0.39	0.60	0.71	1.78
356	Rassf5	−0.07	0.07	−0.34	0.76	0.49	2.30
357	Lims2	−0.20	−0.19	−0.23	1.16	0.95	2.63
358	Map3k6	−0.09	0.04	−0.16	1.60	0.57	1.89
359	Tubb2a	−0.19	−0.17	−0.27	1.24	0.63	0.57
360	Palld	0.04	−0.09	0.10	1.72	−0.14	1.62
361	Eif2s2	−0.14	−0.28	−0.22	0.51	0.63	1.66
362	Rhoc	−0.17	−0.21	0.28	0.91	0.16	2.41
363	R3hdml	−0.05	−0.16	0.05	0.49	1.58	1.89
364	Fetub	−0.15	0.27	−0.29	−0.13	3.72	0.78
365	Dpy19l1	0.12	−0.20	−0.01	0.01	4.21	−0.07
366	Crlf3	−0.21	−0.23	0.05	0.29	2.20	0.25
367	Etnk2	−0.03	−0.03	0.02	−0.02	0.83	4.63
368	Sgk1	−0.29	−0.03	−0.20	0.24	0.03	2.33

･･･1 ＜ Normalized Data
･･･0.5 ＜ Normalized Data ≦ 1
･･･0.3 ＜ Normalized Data ≦ 0.5
･･･-0.3 ＜ Normalized Data ≦ 0.3
･･･-0.5 ＜ Normalized Data ≦ -0.3
･･･-1 ＜ Normalized Data ≦ -0.5
･･･Normalized Data ≦ -1
･･･ Not detected or compromised

List of genes preferentially expressed in sublines with high responsiveness and already reported to be regulators of insulin secretion. P means that the gene is reported to be a positive regulator, and N means reported to be a negative regulator.

Genes whose expression is higher in sublines with high glucose-responsiveness

			H1	H2	H3	ML1	ML2	ML3
**Adora1**	Johansson SM, et al. Biochem Pharmacol. 74, 1628-1635 (2007)	N	0.41	0.39	−0.22	−1.00	−2.27	0.22
**Agt**	Ramrachya RD, et al. Diabetologia. 49, 321-331 (2006)	P	0.01	0.86	1.69	−1.42	−0.44	−0.71
**Cacna1c**	Schulla V, et al. EMBO J. 22, 3844-3854 (2003)	P	0.60	−0.02	0.05	−0.91	−0.83	−0.86
**Cacna1d**	Reinbothe TM, et al. Diabetologia. 56, 340-349 (2013)	P	0.73	0.88	−0.08	−0.17	−0.03	−1.59
**Cd44**	Kobayashi N, et al. Sci Report. 8, 2785 (2018)	P	1.59	−0.05	0.06	−0.41	−1.17	0.05
**Chrnb4**	Ganic E, et al. Cell Rep. 14, 1991-2002 (2016)	P	1.29	0.27	0.01	−1.09	−0.01	−2.73
**Dagla**	Powell DR, et al. Front Endocrinol. 6, 86 (2015)	P	0.91	1.05	0.00	−0.75	−0.00	−0.66
**Esyt1**	Xie B, et al. FASEB J. 33, 4716–4728 (2019)	N	0.12	0.32	0.06	−0.54	−0.50	−1.64
**Ffar2**	Tang C, et al. Nat Med. 21, 173-177 (2015)	N	0.92	0.67	0.90	−0.61	−0.23	−1.30
**Gem**	Gunton JE, et al. PLoS One. 7, e39462 (2012)	P	0.84	0.62	0.12	−0.12	−0.56	−0.90
**Glra1**	Hall E, et al. Mol Cell Endocrinol 472, 57-67 (2018)	P	1.52	1.43	0.12	−0.12	−0.79	−0.72
**Gpr142**	Lin HV, et al. PloS ONE 11, e0157298 (2016)	P	1.39	2.49	−0.12	−3.57	0.12	−2.97
**Gprc5c**	Amisten S, et al. Endocr J. 63, 325-338 (2017)	P	0.58	1.10	0.86	−1.37	−0.58	−0.70
**Hvcn1**	Pang H, et al. J Biol Chem. 295, 3601-3613 (2020)	P	3.23	2.82	−0.20	−0.46	−1.00	−2.66
**Igf1r**	Kulkarni R., et al. Nat Genet. 38, 583-588 (2006)	P	0.09	0.43	−0.06	−2.35	−1.30	−0.23
**Kcnb1**	Fu J, et al. Diabetes. 66, 1890-1900 (2017)	P	0.33	0.36	0.34	−1.09	−0.83	−0.18
**Nnt**	Santos LRB, et al. Mol Metab. 6, 535-547 (2017)	P	0.73	0.34	0.05	−1.79	−0.05	−1.01
**Pcsk9**	Mbikay M, et al. FEBS Lett. 584, 701-706 (2010)	P	1.62	1.02	0.03	−0.03	−0.68	−0.35
**Prkcd**	J Biol Chem. 282, 2707-2716 (2007)	P	1.23	0.44	0.44	−1.77	−1.13	−0.85
**Ptger4**	Wu C-T, et al. Gene Develop. 35, 1243–1255 (2021)	P	1.73	1.40	−0.27	0.27	−0.33	−0.41
**Ptprn2**	Cai T, et al. Diabetologia. 54, 2347-2357 (2011)	P	1.23	1.13	0.05	−0.40	−0.05	−0.73
**Rab43**	Rhee M, et al. Sci Report. 6, 23960 (2016)	P	1.16	0.51	−0.07	−0.71	−0.62	−0.64
**Rgs16**	Vivot K, et al. Mol Metab. 5, 988-996 (2016)	P	0.88	1.58	−0.29	0.10	−1.87	
**Rorb**	Taneera J, et al. Islets. 11, 10-20 (2019)	P	2.79	1.80	0.01	−0.27		
**Slc6a19**	Broer A, et al. J Biol Chem. 286, 26638-26651 (2011)	P	1.17	0.64	0.57	0.23	−0.65	−0.23
**Sphk1**	Hasan NM, et al. Arch Biochem Biophy. 18, 23-30 (2012)	P	0.39	2.48	0.05	−1.74	−2.62	−0.05
**Sytl4**	Wang H, et al. J Biol Chem. 286, 32244-32250 (2011)	P	0.87	0.04	−0.20	−0.03	−0.74	−2.00
**Tagln2**	Li J, et al. J Proteome Res. 14, 4635-4646 (2015)	P	0.58	0.46	−0.04	−2.69	−0.43	−0.57
**Tmem37**	Solimena M, et al. Diabetologia. 61, 641-657 (2018)	P	2.54	0.71	0.13	−0.09		
**Ucn3**	van der Meulen T, et al. Nat Med. 21, 769-776 (2015)	N	3.06	2.12	−0.01	−3.17	−1.77	−6.10

List of genes preferentially expressed in sublines with reduced-responsiveness and already reported to be regulators of insulin secretion. P means that the gene is reported to be a positive regulator, and N means reported to be a negative regulator.

Genes whose expression is higher in sublines with mildly low glucose-responsiveness			H1	H2	H3	ML1	ML2	ML3
Adcy5	Hodson DJ, et al. Diabetes. 63, 3009-3021 (2014)	P			−1.25	0.15	1.82	−0.15
Aldh1b1	Anastasiou V, et al. Diabetologia. 59, 139–150 (2016)	P	−0.74	0.07	−0.38	0.97	−0.07	2.05
Arrb1	Barella LF, et al. Diabetologia. 57, 1899-1910 (2014)	P	−2.21	−1.84	0.01	0.66	−0.30	−0.01
Atp9a	Ansari IH, et al. J Biol Chem. 290, 23110-23123 (2015)	P	−0.77	−0.95	−0.68	0.80	0.59	0.18
Cacna1g	Yu J, et al. Proc Natl Acad Sci USA. 117, 448-453 (2020)	N	−1.52	−1.58	0.24	0.37	0.44	−0.24
Calb1	Christensen GL, et al. Diabetologia. 58, 1282-1290 (2015)	N	−0.10	−0.61	0.10	0.37	0.39	1.89
Cbln1	Strowski MZ, et al. Regul Pept. 157, 19–24 (2009)	N				0.31	0.19	0.84
Ctf1	Jimenez-Gonzalez M, et al. Diabetologia. 56, 838-846 (2013)	P	−0.08	−0.11	−0.75	0.85	0.08	1.87
Egln3	Huang M, et al Physiol Rep. 4, e12722 (2016)	P	−2.00	−0.72	0.13	−0.09	0.09	0.67
Etv4	Suriban R, et al. Cell. 163, 1457–1467 (2015)	N	−0.81	−0.22	−0.05	0.05	0.53	1.39
Fhl2	Bacos K, et al. Nature Commun. 7, 11089 (2016)	P		−1.12	−0.32	3.39	0.32	4.02
Foxa1	Gao N, et al. Cell Metab. 6, 267–279 (2007)	N			−3.42	0.29	−0.29	1.95
Gast	Khan D, et al. Pancreas. 47, 190–199 (2018)	P	−0.86	−0.24	−1.02	0.56	0.92	0.47
Gckr	Zhou F, et al. Theranostics. 11, 4825-4838 (2021)	N				0.48	0.49	1.36
Gnas	Taneera J, et al. Gene 715, 144028 (2019)	P	−0.10	−0.36	−2.11	1.00	0.10	−0.24
Hhip	Nchienzia H, et al. Sci Report. 9, 11183 (2019)	N	−0.19	−0.18	−1.51	1.19	1.76	3.14
Hmgb1	Li M, et al. Exp Mol Med. 44, 260-267 (2012)	N	−0.15	−0.19	−1.46	0.82	0.72	−0.28
Kcna4	MacDonald PE, et al. Mol Endocrinol. 15, 1423-1435 (2001)	N	−0.45	−0.17	−0.41	1.04	0.91	0.14
Kiss1r	Chen J, et al. Mol Endocrinol. 28, 1276-1290 (2014)	N	−0.88	−0.18	−0.16	0.83	0.14	0.95
Mapt	FASEB J. 32, 3166–3173 (2018)	P	−1.06	−1.28	−0.16	0.16	0.63	0.86
Nrg4	South JCM, et al. Endocrinology. 154, 2385–2392 (2013)	P			−0.08	0.08	1.69	2.85
P2rx3	Jacques-Silva MC, et al. Proc Natl Acad Sci USA. 107, 6465-6470 (2010)	P	−0.73	−0.15	−1.35	0.70	0.18	0.16
P2ry14	Meister J, et al. J Biol Chem. 289, 23353-23366 (2014)	P			0.04	1.55	1.18	1.46
Park2	Jin H-K et al. Mol Cell Endocrinol. 382, 178–189 (2014)	P	−0.88	−0.61	0.19	0.73	0.75	0.62
Pbx1	Dal P, et al. l Dev Biol. 9, 685494 (2021)	P				0.26	0.34	0.93
Pde8b	Dov A, et al. Endocrinology. 149, 741-748 (2008)	N	−0.39	−0.18	0.06	0.90	1.81	−0.08
Plin5	Trevino MB, et al. Diabetes 64, 1299–1310 (2015)	P	−2.13	−0.28	0.28	2.19	1.04	2.31
Prex1	Thamilselvan V, et al. Cell Physiol Biochem. 54, 1218-1230 (2020)	P	−2.07	−1.58	0.09	−0.00	0.06	0.00
Six3	Bevacqua RJ, et al. Genes Dev. 35, 234-249 (2021)	P	−1.24		0.02	−0.28	1.32	1.54
Slit1	Hsuan Y, et al. Proc Natl Acad Sci. U.S.A. 110, 16480–16485 (2013)	P	−2.03	−1.46	−0.06	0.57	0.42	1.13
Tspan33	Fadista J, et al. Proc Natl Acad Sci USA. 111, 13924-13929 (2014)	P	−0.08	0.08	−0.39	0.60	0.71	1.78
Txnip	Deng W., et al. Endocrine. 70, 526–537 (2020)	N	0.00	−0.00	−0.58	2.81	0.18	0.79
Wnt4	Kurita Y, et al. Kurume Med J. 65, 55-62 (2018)	P	−2.50	−0.05	−1.82	0.41	0.05	0.06

List of preferentially expressed genes in sublines with high glucose-responsiveness (upper) and mildly low responsiveness (lower) and are already reported to be regulators of β-cell stress response or β cell proliferation/survival.

Genes whose expression is higher in sublines with high glucose-responsiveness

		H1	H2	H3	ML1	ML2	ML3
**Anks4b**	Sato Y, et al. J Biol Chem. 287, 23236-23245 (2012)	0.76	0.59	0.27	−0.40	−0.20	−1.40
**Cxcl12 = Sdf1**	Grdovic N, et al. PLoS One. 9, e101172 (2014)	1.26	2.38	0.29	−0.29	−2.46	−1.89
**Dhcr24**	Li Y, et al. J Diabet Res. 3426902 (2020)	1.12	0.91	−0.03	−0.45	−0.61	0.03
**Egfr**	Hakonen E, et al. Diabetologia. 54, 1735–1743 (2011)	1.19	0.87	0.11	−3.64	−3.04	−0.11
**Lefty1**	Kluth O, et al. PLoS Genet. 11, e1005506 (2015)	4.43	4.33	2.29			
**Pax4**	Zhang T, et al. Int J Mol Sci. 20, 6171 (2019)	0.34	1.01	0.69	−0.69		−3.55
**Ripply3**	Osipovich AB, et al. Development. 141, 2939-2949 (2014)	0.28	0.27	−0.11	−2.25	−0.75	−3.32
**Usp18**	Santin I, et al. Cell Death Dis. 3, e419 (2012)	1.10	0.45	0.18	−0.58	−0.36	−0.84

Genes whose expression is higher in sublines with mildly low glucose-responsiveness

		H1	H2	H3	ML1	ML2	ML3
**C4bp**	Sjolander J, et al. J Biol Chem. 287, 10824-10833 (2012)				3.86	0.51	6.84
**Cdkn1c**	Asahara S-I, et al. Proc Natl Acad Sci U S A. 112, 8332-8337 (2015)	−0.92	−1.27	0.24	0.14	1.11	−0.11
**Clec11a**	Shi R, et al. Exp Cell Res 384, 111613 (2019)	−1.65	−1.03	−0.00	0.00	0.48	1.04
**Ctsh**	Floyel T, et al. Proc Natl Acad Sci USA. 111, 10305-10310 (2014)	−0.30	−0.13	−0.05	0.65	0.32	3.85
**Lats2**	Guo R, et al. Am J Physiol Endocrinol Metab. 318, E430–E439 (2020)	0.20	−2.56	−0.20	0.43	0.60	0.82
**Stat4**	Weaver JR, et al. PLoS One. 10, e0142735 (2015)				1.13	0.32	0.32
**Tspan2**	Hwang I-H, et al. FASEB J. 30, 3107–3116 (2016)	−0.57	−0.58	−0.42	0.60	0.18	0.75
**Uchl1**	Costes S, et al. Diabetes. 55, 1223-1231 (2006)	−4.12	−3.85	−1.56	2.07	1.56	2.87

Insulin secretory characteristics (GSIS) and insulin content of MIN6 cell sublines used in previous studies and ours

	Sublines	GSIS	Cellular Insulin content
**Minami et al. (ref. 7)**	MIN6-m9 (high responder)	Around 10-fold	not described
	MIN6-m14 (poor responder)	No response	not described
**Lilla et al. (ref. 8)**	B1 cells (High responder)	Around 10-fold	4.0 mg/10^6 cells
	C3 cells (poor responder)	No response	4.5 mg/10^6 cells
**O'Driscoll et al. (ref. 9)**	MIN6 (Low passage)	5 to 6-fold	3.73 pmol/ml/mg protein
	MIN6 (High passage)	No response	3.57 pmol/ml/mg protein
**Yamato et al. (ref. 10)**	Pr-Low passage	Around 7-fold	15 mg insulin/mg protein
	Cl4-Low passage	Around 10-fold	6 mg insulin/mg protein
	Cl4-High passage	Around 10-fold	8 mg insulin/mg protein
	Pr-High passage	No response	2 mg insulin/mg protein
**Tanaka et al. (present study)**	High responder 1	10.7-fold	17.0 mg insulin/mg protein
	High responder 2	10.2-fold	16.5 mg insulin/mg protein
	High responder 3	11.4-fold	17.3 mg insulin/mg protein
	Mildly low responder 1	4.7-fold	17.5 mg insulin/mg protein
	Mildly low responder 2	4.8-fold	18.6 mg insulin/mg protein
	Mildly low responder 3	2.4-fold	16.4 mg insulin/mg protein

List of primers used for cDNAs cloning

These primers were also used for overexpression validations by RT-PCR.

		Forward primers	Reverse primers
Acot11		5'-GTACAGGTCGACAGCACTTGGGTAAAACCCGATG-3'	5'-GTCTAGGCGGCCGCTTTGTTCCACGCACTGTGTAAG-3'
Adh1		5'-CCCTCGTAAAGTCGACCACCATGAGCACTGCGGGAAAAGTGAT-3'	5'-CCTGAGGAGTGAATTCAGAAAGTCAGGACGGTACGGATG-3'
Agpat2		5'-CCCTCGTAAAGTCGACCACCATGGACCCGTGGCCAT-3'	5'-CCTGAGGAGTGAATTCTACTGGGCTGGCAAGACCC-3'
Akr1c14		5'-CCCTCGTAAAGTCGACCACCATGAATTCTGTATCCCCACGTGTG-3'	5'-CCTGAGGAGTGAATTCTCTTCTGATAGAAATGCTGGCAAAGG-3'
Apoe		5'-GATGGCTGGTCGACCACCATGAAGGCTCTGTGGGCCGT-3'	5'-GTCTAGTGGAATTCATTGATTCTCCTGGGCCACTGG-3'
Bricd5		5'-CCCTCGTAAAGTCGACCACCATGTACAGCTCTCCCAAGCCG-3'	5'-CCTGAGGAGTGAATTCGTAGGGGGTCTTAGTCTGGGAGGTAAT-3'
Camk1d		5'-CCCTCGTAAAGTCGACCACCATGGCCCGGGAGAACG-3'	5'-CCTGAGGAGTGAATTCACTTGCTTCCAGTGTGCCCT-3'
Car15		5'-GATGGCTGGTCGACCACCATGTGGGCCCTGGACTTCTT-3'	5'-GTCTAGTGGCGGCCGCGAATCATTGCTGGACCTAGGGACC-3'
Car5b		5'-GATGGCTGGTCGACCACCATGGCTGTGATGAATCACCTGAGAG-3'	5'-GTCTAGTGACTAGTCAAGATGCTGCTGAAGAATGCTGTC-3'
Ccdc8		5'-CCCTCGTAAAGTCGACCACCATGCTGCAGATTGGGGAGGATG-3'	5'-CCTGAGGAGTGAATTCCCTTACAGCTGTTCTTCTTGCTCT-3'
Ccnb1ip1		5'-GATGGCTGGTCGACCACCATGTCTTTGTGTGAAGACATGCTG-3'	5'-GTCTAGTGGCGGCCGCGAAGAGAACTCAGGGATGCAGGA-3'
Crip1		5'-GATGGCTGGTCGACAGAGCCTGCAACCTACTTCCTTCTA-3'	5'-GTCTAGAGGAATTCTACTTGAAAGTGTGGCTCTCAGC-3'
Cxcl16		5'-GTACAGGTCGACCACCATGAGGCGGGGTTTGGA-3'	5'-GTCTAGGAATTCGCTAGGGTCTTGGTTCAACAGG-3'
Cyct		5'-GTCGACCACCATGGGAGATGCTGAAGCAGGC-3'	5'-GAATTCACTTCATGAGGATGTGGCCTGTTTTAAATACT-3'
Dab1		5'-GCTGGTCGACCACCATGTCAACTGAGACAGAACTTCAAGT-3'	5'-GTCTAGAGGAATTCTAGCTACCGTCTTGTGGAC-3'
Dbn1		5'-CCCTCGTAAAGTCGACCACCATGGCCGGCGTCAGCTT-3'	5'-CCTGAGGAGTGAATTCTAATCACCACCCTCGAAGCCCT-3'
Dclk2		5'-GTACAGGTCGACCACCATGGCCAGCACAAGGAGCATT-3'	5'-GTCTAGGCGGCCGCCTGTGTACACAGGGTTCAGTC-3'
Dhdh		5'-GATGGCTGGTCGACGAAGACGTGCAAAATGGCGCT-3'	5'-GTCTAGTGGCGGCCGCTCAGCGTTTATCCTGGGGGAAG-3'
Dpysl3		5'-GATGGCTGGTCGACCAGAATCGCCACCATGTCCT-3'	5'-GTCTAGTGGCGGCCGCGGGAGGGCTTAACTCAGGGATG-3'
Emilin1		5'-GATGGCTGACTAGTCAGCAAGGAACATTTCACCATGGC-3'	5'-GTCTAGTGGAATTCTACACCTGTTCAAGCTCTGTGTC-3'
Eps8l1		5'-CCCTCGTAAAGTCGACCACCATGAGCACCACCACAG-3'	5'-CCTGAGGAGTGAATTCAAATGACTTCTGTTTTGGTCTCGCCTTC-3'
Eya2		5'-CCCTCGTAAAGTCGACCACCATGTTAGAAGTGGTGACCTCACCC-3'	5'-CCTGAGGAGTGAATTCTATAGATACTCCAGTTCCAGGGCATG-3'
Fam107b		5'-CCCTCGTAAAGTCGACCACCATGGCTGAGCCAGACTACATAGAAG-3'	5'-CCTGAGGAGTGAATTCTAGGACTCCTGGGCCTGAG-3'
Fam126a		5'-GATGCTGGTCGACCACCATGTTCACCTCAGAGATAGGAGTTGTGG-3'	5'-GTCTAGTGGAATTCTTACTCTGCAGACAGAGTGACGCT-3'
Fam151a		5'-GATGGCTGGTCGACCACCATGTCCTGCAAGAAATGGTGCTCC-3'	5'-GTCTAGAGGAATTCACCTTGACTCCCCAGGGAA-3'
Fbln7		5'-GATGGCTGGTCGACAAGGCTGTGAGTGGCAAGATG-3'	5'-GTCTAGTGGAATTCGGCATCCTCAGAAGTCATAGCGA-3'
H2-Eb		5'-CCCTCGTAAAGTCGACCACCATGGTGTGGCTCCCCAGAG-3'	5'-CCTGAGGAGTGAATTCATCTCAGCTCAGGAGTCCTGTTGG-3'
Hck		5'-GATGGCTGGTCGACTGTAGCCCGCAAGTCTTCGTC-3'	5'-GTCTAGAGGAATTCAAGGCTGCTGCTGATACTGGC-3'
Hs2st1		5'-CCCTCGTAAAGTCGACCACCATGGGGCTCCTCAGGATTATGATG-3'	5'-CCTGAGGAGTGAATTCACCTGCGCTCAGTTCGACTTAG-3'
Igfbp4		5'-GATGGCTGGTCGACCACCATGCTGCCCTTCGGCCT-3'	5'-GTCTAGTGGAATTCAGGTCTCACTCTTGGAAGCTGTCA-3'
Il11ra1		5'-GATGTGGTCGACCACCATGAGCAGCAGCTGCTCAGG-3'	5'-TCGCATGCGGCCGCTCAGCTGAAGTTCTCTGGGGTCC-3'
Kcnj12		5'-GATGGCTGGTCGACCACCATGACCGCAGCCAGTCGGG-3'	5'-GTCTAGAGGCGGCCGCTCAAATCTCCGACTCCCGTCTG-3'
Lhfpl2		5'-CCCTCGTAAAGTCGACCACCATGTGTCATGTCATTGTCACCTGC-3'	5'-CCTGAGGAGTGAATTCTTAAAGGAGGCAGACGAGGTTTTTCC-3'
Lims2		5'-CCCTCGTAAAGTCGACCACCATGACGGGAAGCAACATG-3'	5'-CCTGAGGAGTGAATTCCTTTAGAGTGAGTTGACGTCCACAGAC-3'
Mgst2		5'-CCCTCGTAAAGTCGACCACCATGGCCGGGGATTCAAGCC-3'	5'-CCTGAGGAGTGAATTCTTAGAAGGGCTTCCTCAGTTTCTTGGC-3'
Mppe1		5'-CCCTCGTAAAGTCGACCACCATGGCTCTGGTCAGATGGGG-3'	5'-CCTGAGGAGTGAATTCGGCTCTTCATCTCCTCATGTGC-3'
Msn		5'-CCCTCGTAAAGTCGACCACCATGCCGAAGACGATCA-3'	5'-CCTGAGGAGTGAATTCGCCCACTACATGGACTCAAACTC-3'
Mtap7d3		5'-CCCTCGTAAAGTCGACCACCATGGCGGACCCCACTTTTGC-3'	5'-CCTGAGGAGTGAATTCAAGGCCCCACAGGATTTACACATC-3'
Necab2		5'-CCCTCGTAAAGTCGACCACCATGTGCGAGCGGGCGGCG-3'	5'-CCTGAGGAGTGAATTCTTAACAGGGAGGGGTCAGTCTCG-3'
Nipsnap1		5'-CCCTCGTAAAGTCGACCACCATGGCTCCGCGGTTGTG-3'	5'-CCTGAGGAGTGAATTCACTGGAGAGGAGAAATCTTCAGAGG-3'
Nptx2		5'-CCCTCGTAAAGTCGACCACCATGCTGGCGCTGCTGACC-3'	5'-CCTGAGGAGTGAATTCTACAAGTCCAGGAGCCGCTCTT-3'
Plin3		5'-CCCTCGTAAAGTCGACCACCATGTCTAGCAATGGTACAGATGCG-3'	5'-CCTGAGGAGTGAATTCTACTTCCCTTCAGGGGTTTTCTCTGTG-3'
Prickle3		5'-CCCTCGTAAAGTCGACCACCATGTTCGCGCGTGGGT-3'	5'-CCTGAGGAGTGAATTCCTTTAAGCCACGATGCAGTTCTTGTC-3'
Prss8		5'-CCCTCGTAAAGTCGACCACCATGGCCCTAAGGGTGGG-3'	5'-CCTGAGGAGTGAATTCAAGCCAAAGACAAGAGGCCCA-3'
Pter		5'-AACCGTCGACCACCATGTCTTCCTTAAGTGG-3'	5'-CCTTGAATTCAGCCATCCTATTTAAAAGTCAGCCATTGTTTAGG-3'
Rab19		5'-GATGGCTGGTCGACCACCATGCAGTTCTCCAGCTCATCCAGGACATC-3'	5'-GTCTAGTGGAATTCAACAAGTACAGCGGGTGCTCTCATTGG-3'
Rab9b		5'-CCCTCGTAAAGTCGACCACCATGAGTGGGAAATCCCTTCTCTT-3'	5'-CCTGAGGAGTGAATTCCCTACCTCTAACAACAAGAAGAACT-3'
Rasl10a		5'-GATGGCTGGTCGACGGGAGCGCGGGCCAGCCATG-3'	5'-GTCTAGTGGAATTCTGCTTTCAAGAGATTTCCCTGTCCAATC-3'
Rftn1		5'-GATGGCTGGTCGACCACCATGGGTTGCAGTTTGAACAAGC-3'	5'-GTCTAGAGGAATTCGGACTCAATTTGCTTCTGTTGG-3'
Rnf135		5'-GATGGCTTGTCGACCACCATGGCGGCCGTTTGTTCTG-3'	5'-GTCTAGTGGAATTCGGGAAGCTCATGTGTTTAGCTGC-3'
Scnn1b		5'-CCCTCGTAAAGTCGACCACCATGCCAGTGAAGAAGTACC-3'	5'-CCTGAGGAGTGAATTCTCTAGATGGCCTCCACCTCACTG-3'
Sec14l3		5'-CCCTCGTAAAGTCGACCACCATGAGTGGCCGAGTTGG-3'	5'-CCTGAGGAGTGAATTCTAGACAGGGGTGAGCTCCTCATC-3'
Sh3rf2		5'-GATGGCTGGTCGACCACCATGGATGATTTGACGTTACTTGATCTCCTGG-3'	5'-GTCTAGTGGAATTCCCGAAGAATCATTTGCTGGGAAACAA-3'
Slc18b1		5'-CCCTCGTAAAGTCGACCACCATGGACGAGGCGGGCTC-3'	5'-CCTGAGGAGTGAATTCTAGGTGTCATTGGGCAAGAGAGCA-3'

		Forward primers	Reverse primers
Slc37a2		5'-CCCTCGTAAAGTCGACCACCATGCGGTCCTCCCTGGCTC-3'	5'-CCTGAGGAGTGAATTCGGCCTCAAATTTGTTTGTACCCACTGC-3'
Slc39a11		5'-CCCTCGTAAAGTCGACCACCATGCTCCAAGGTTACAGCTCCG-3'	5'-CCTGAGGAGTGAATTCTAGCCGAGGCCCACATCAAG-3'
Slc41a3		5'-CCCTCGTAAAGTCGACCATGGTCGTCACTCAGCTCAGC-3'	5'-CCTGAGGAGTGAATTCGCTCAGTCAGGGAGGCACAG-3'
Slx		5'-GATGGCTGGTCGACAGGGTTGTTGGACAGTTAATCGAG-3'	5'-GTCTAGTGGAATTCGGGAAAGGAGAAGAGTACTTCAGAGTATG-3'
Smarcd3		5'-CCCTCGTAAAGTCGACCACCATGGCCGCGGACGAAGTT-3'	5'-CCTGAGGAGTGAATTCCTAGGTGTTGCGCACGAC-3'
Smtnl2		5'-GATGGCTGGTCGACAAGGAGCTGCGGATCTCTCAA-3'	5'-GTCTAGTGGAATTCTTACTCAAAGCGACGAAGGTGGTT-3'
Sowaha		5'-CCCTCGTAAAGTCGACAGACACCGAGAATGGCGCTG-3'	5'-CCTGAGGAGTGAATTCAGGTTGGAGGGGGCAGA-3'
Sox11		5'-GATGGCTGGTCGACCACCATGGTGCAGCAGGCCGA-3'	5'-GTCTAGAGGAATTCTCAATACGTGAACACCAGGTCGGAG-3'
Srgap1		5'-CCCTCGTAAAGTCGACCACCATGTCCACCCCGAGCAGATTCAA-3'	5'-CCTGAGGAGTGAATTCTTACATCGTGCAAGACTTGTCTGTGGG-3'
St6gal1		5'-CCCTCGTAAAGTCGACCACCATGATTCATACCAACTTGAAGAGAA-3'	5'-CCTGAGGAGTGAATTCAACAGCGATTGTTCCG-3'
Stmn4		5'-GATGGCTGGTCGACCACCATGACTCTTGCAGCCTATAAGGAGAAGATGA-3'	5'-GTCTAGTGAATTCGCCTCTGGGCTTTACCTGGAG-3'
Sult1c2		5'-GTACAGGTCGACCACCATGGCCTTGACCCCAGAAC-3'	5'-GTCTAGGAATTCGATCAGAGTTCCATGGAGAAGTTCAGAG-3'
Sult4a1		5'-CCCTCGTAAAGTCGACCACCATGGCGGAGAGCGAAGCG-3'	5'-CCTGAGGAGTGAATTCCGTGAGCATGCAAGTTCTCGG-3'
Svop		5'-CCCTCGTAAAGTCGACCACCATGGAGGAGGATCTGTTCCAGCTC-3'	5'-CCTGAGGAGTGAATTCTACTCCTGAGAGCCAGAGTCCG-3'
Tgm5		5'-GTACAGCTCGAGAGCCAGAAGGAGCCACCATG-3'	5'-GTCTAGGAATTCAGCCTCTGTCTCAGAGTTTATAAGCC-3'
Tmcc3		5'-GATGGCTCGTCGACGTTGTCAGTCTAAGCGCAGACATG-3'	5'-GTCTAGTGACTAGTGGCTCATCTCGGGATGATTATCCT-3'
Tmem171		5'-GTACAGGTCGACCACCATGTCTTCTGTAGGAACTGCTGAG-3'	5'-GTCTAGGAATTCTTGAGTCCATAGCTCATGGCGG-3'
Tmem200a		5'-CCCTCGTAAAGTCGACCACCATGATAGCCACTGGTGGGGTCAT-3'	5'-CCTGAGGAGTGAATTCACTAAAACCTTGTCTCAGAAGTTCCTCGC-3'
Tnk1		5'-GATGGCTGGTCGACCACCATGCTCCCTGAAGCCAGTTC-3'	5'-GTCTAGTGGAATTCAGGACCGGGCTAGGATG-3'
Tspan15		5'-CCCTCGTAAAGTCGACCACCATGCCTCGCGGGGACTCG-3'	5'-CCTGAGGAGTGAATTCTAATCGGGATAGCACAGGCAGCAT-3'
Tspan18		5'-GATGGCTGGTCGACTGCCCAGGTGAAGAACCATG-3'	5'-GTCTAGTGGCGGCCGCTCTTCAGGCCAAGGCCTACTG-3'
Tspan2		5'-CCCTCGTAAAGTCGACCACCATGGGGCGTTTTCGCGG-3'	5'-CCTGAGGAGTGAATTCTCAGATCACATCGCGTGAGTTCCG-3'
Vil1		5'-CCCTCGTAAAGTCGACCACCATGACTAAACTGAATGCCCAAG-3'	5'-CCTGAGGAGTGAATTCAGCCAGAGAGCTTCAATTCTCAAAACAG-3'
Wdr16		5'-GATGGCTGTCGACCACCATGGAAGAACAAGTTTTACCCGAGCTGG-3'	5'-GTCTAGTGACTAGTCGGAGATCCAGAGCCCATCAC-3'
Wipf1		5'-CCCTCGTAAAGTCGACTTAACTACCCAAGATGCCTGTCCCT-3'	5'-CCTGAGGAGTGAATTCTAGAACGGAGCAGGCAGAGATCA-3'
Xlr		5'-GTACAGGTCGACCACCATGGAAAACTGGGACTTGTCAAGTG-3'	5'-GTCTAGGAATTCAACTGGTTTCTGCTTCTTTCGG-3'
Zfp105		5'-GTACAGGTCGACCACCATGACTACAGAATTGAAAGAGACCATGGG-3'	5'-GTCTAGGCGGCCGCTATTCAACAAGATGGGTTCTGTGATG-3'
Zfp365		5'-CCCTCGTAAAGTCGACCACCATGCAACAGACGACTTTTGAGGA-3'	5'-CCTGAGGAGTGAATTCACCCAAGCTCTAGATGATATTCACG-3'
Zmynd15		5'-GAGAAGGGTCGACCACCATGGAGTTTGTGTCTGGATACCG-3'	5'-AGTAGCCCCTTGCGGCCGCTGGGCCTTGGAATGTTTTCCT-3'

For all forward primers, the Kozak consensus sequence is added to the ATG initial codon. At the initial stage of this study, cDNAs have been cloned by RT-PCR, digested with SalI-EcoRI and ligated to our plasmids pF3BsdTreGFwr or pF3HygTreGFwr. At the later stage, cDNAs have been clones using primers compatible to the InFusion technology. Thus primers are attached with CCTCGTAAAGTCGA and CCTGACGAGTGAATT sequences to 5’ and 3’ primers respectively.

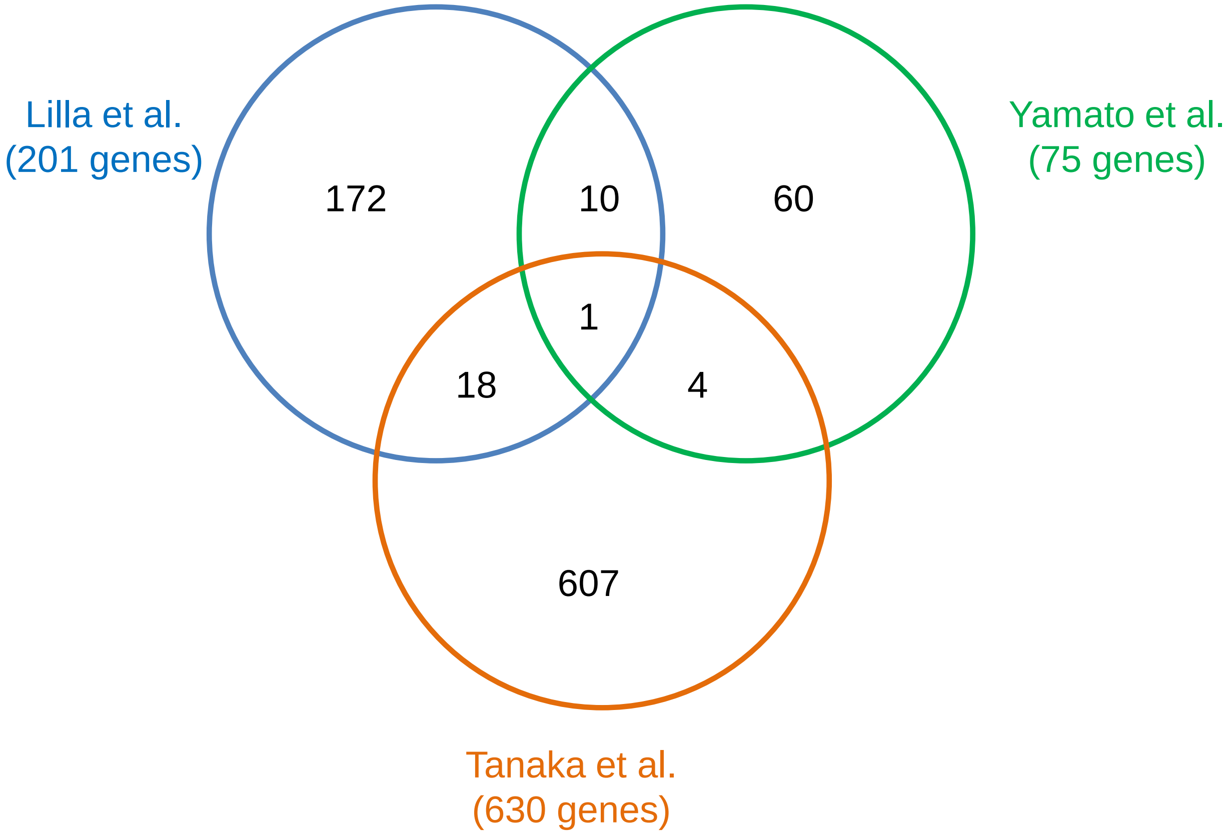

One gene common in three studies is Sox11.

Common 18 genes in Lilla *et al*. and the present studies are *Abcd4, Agt, Cldn7, Crip1, Ctsh, Etv4, Fgf12, Folr1, Gast, Igfbp4, Man1a, Mapt, Mybph, Prkcb, Prom1, St6galnac5, Tenm4,* and *Wnt4*.

Common 4 genes in Yamato *et al*. and ours are *Car2, Cdhr1, Edile,* and *Tmcc3*.

Common 10 genes in Lilla et al. and Yamato *et al*. are *Blnk, Cd200, Enapep, Fgfr2, Pcdh7, Perp, Pparg, Ppgr, Stmn2,* and *Zfp185.*

Uncropped gel images for RT-PCR analysis of overexpression validation in Figure 2

Uncropped gel images used in Figure 2a

Prss8 cell RNA

Prss8 *GAPDH*

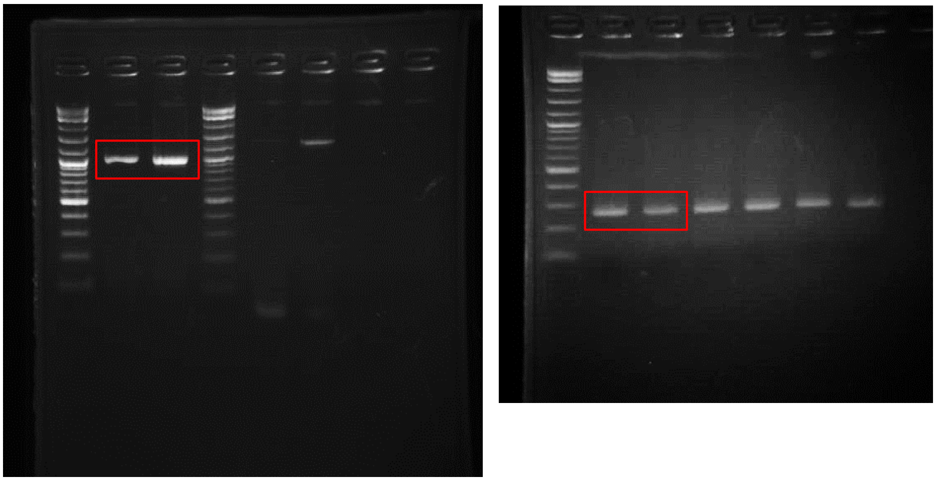

Uncropped gel images used in Figure 2b

Pter cell RNA

Pter *GAPDH*

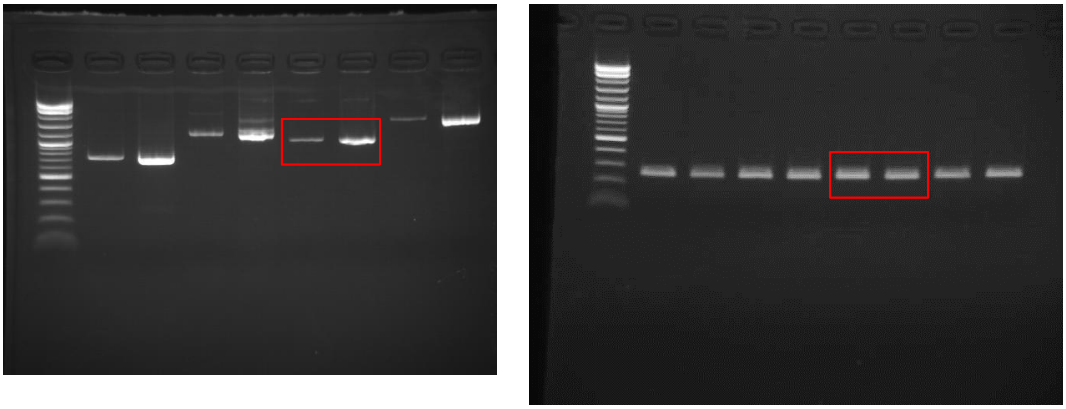

Uncropped gel images for RT-PCR analysis of overexpression validation in Figure 2

Uncropped gel images used in Figure 2c

Dab1 cell RNA

Dab1

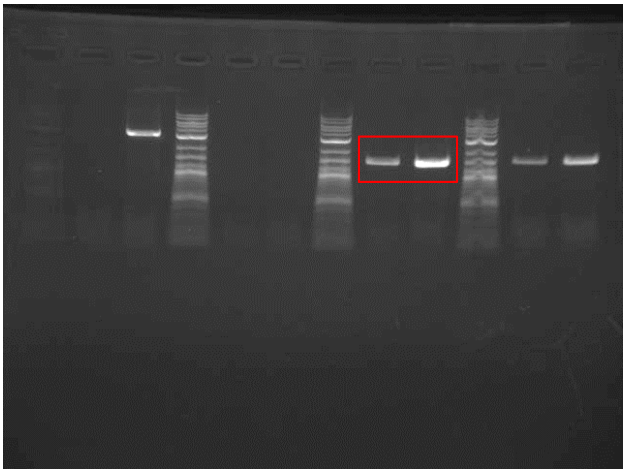

GAPDH

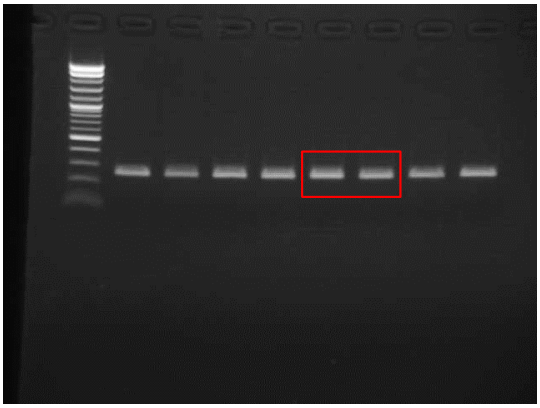

Uncropped gel images used in Figure 2d

Sox11 cell RNA

Sox11

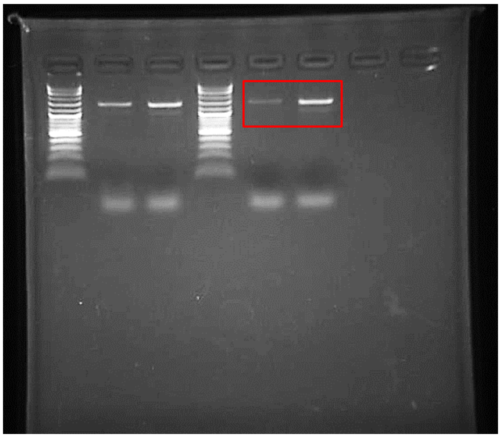

GAPDH

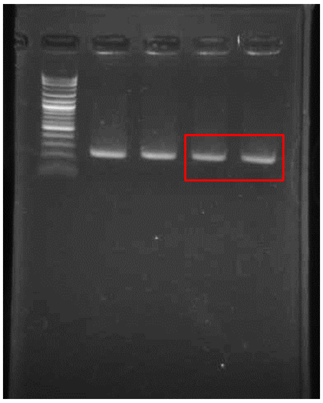

Uncropped Western blot images used in Figure 4b

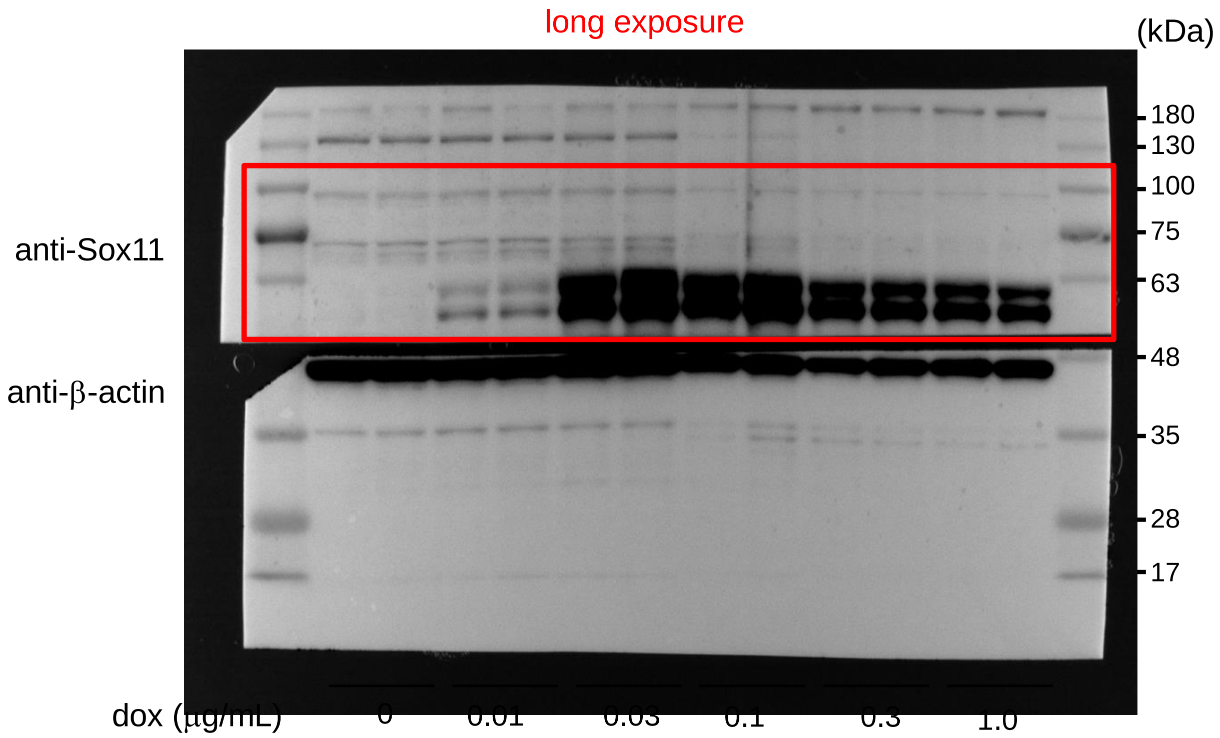

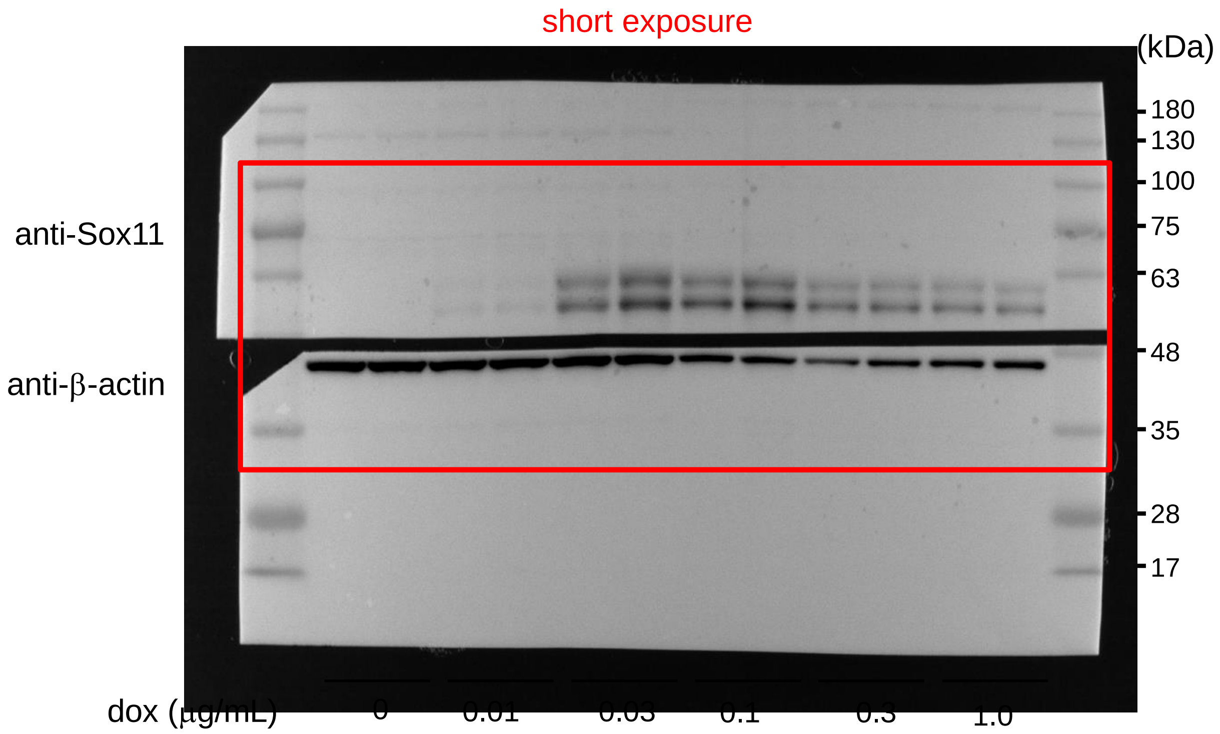

Uncropped Western blot images used in Figure 5

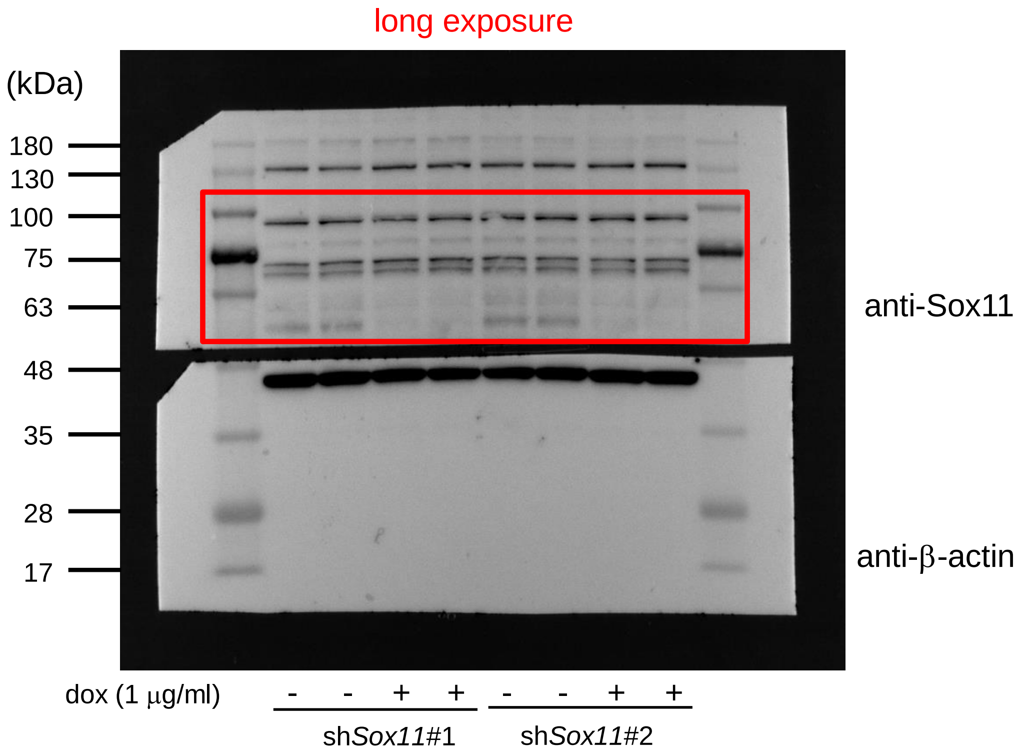

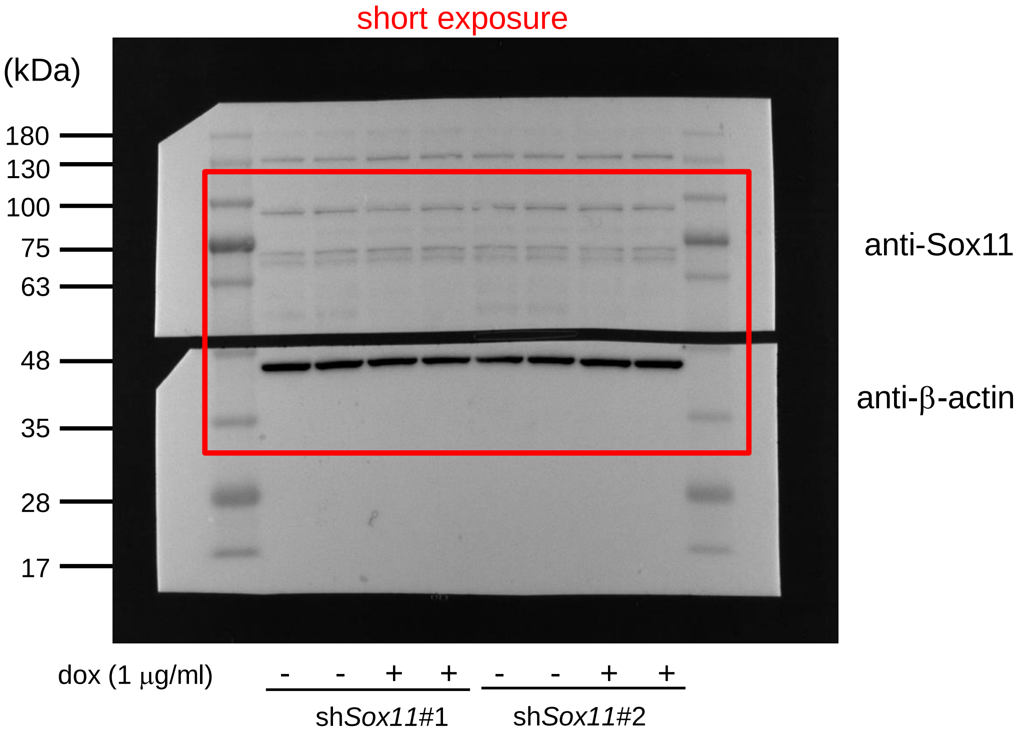

Uncropped Western blot images used in Figure 4d

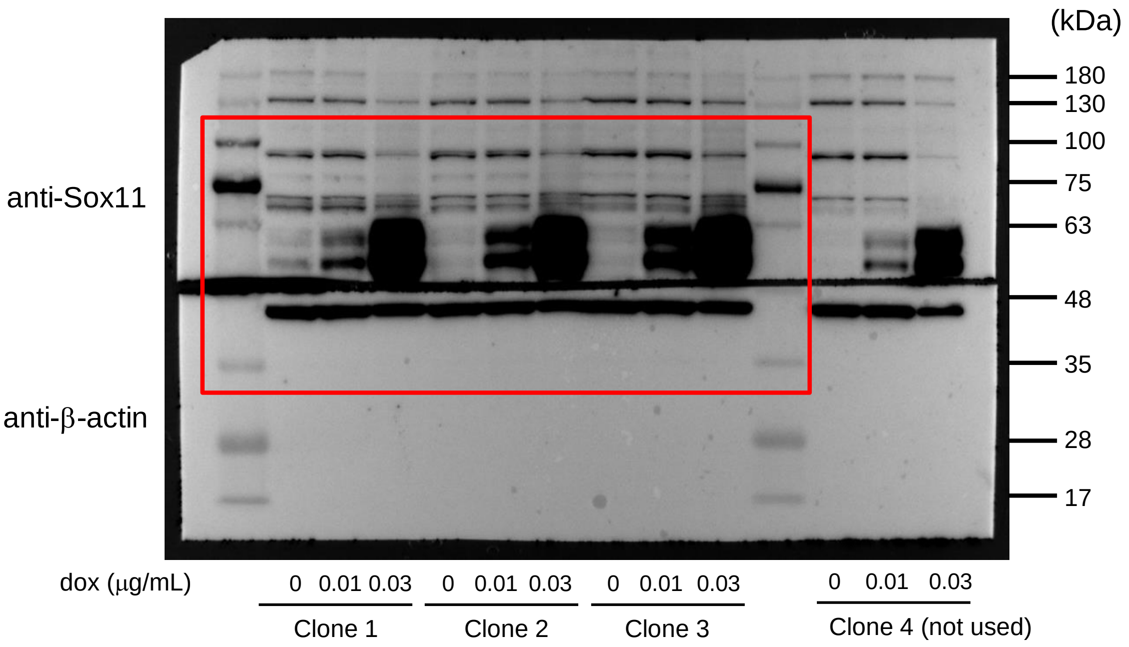

Uncropped Western blot images used in Figure 5e

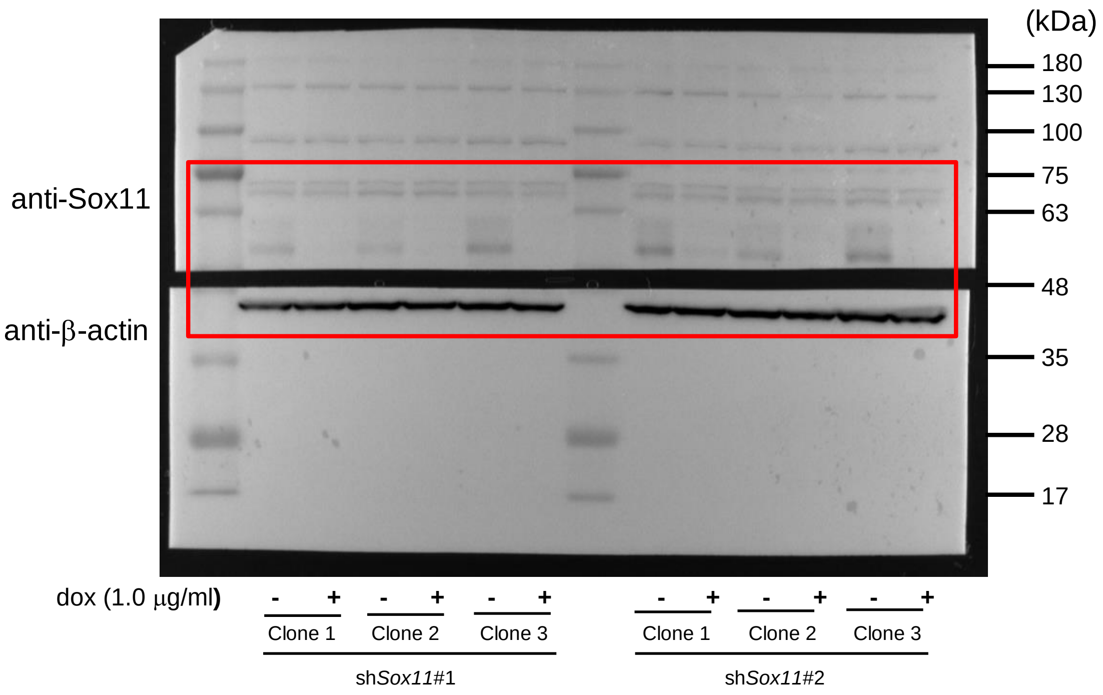

Supplement: Supplementary file 1 — Supplementary Information 1. [file 41598_2023_32589_MOESM1_ESM.pdf]
